# SREBP1 induction mediates long-term statins therapy related myocardial lipid peroxidation and lipid deposition in TIIDM mice

**DOI:** 10.1016/j.redox.2024.103412

**Published:** 2024-10-28

**Authors:** Tong-sheng Huang, Teng Wu, Xin-lu Fu, Hong-lin Ren, Xiao-dan He, Ding-hao Zheng, Jing Tan, Cong-hui Shen, Shi-jie Xiong, Jiang Qian, Yan Zou, Jun-hong Wan, Yuan-jun Ji, Meng-ying Liu, Yan-di Wu, Xing-hui Li, Hui Li, Kai Zheng, Xiao-feng Yang, Hong Wang, Meng Ren, Wei-bin Cai

**Affiliations:** a Guangdong Engineering & Technology Research Center for Disease-Model Animals, Laboratory Animal Center, Guangzhou, 510080, Guangdong, PR China; b Department of Biochemistry, Zhongshan School of Medicine, Sun Yat-sen University, Guangzhou, 510080, Guangdong, PR China; c Department of Endocrinology, Sun Yat-sen Memorial Hospital, Sun Yat-sen University, Guangzhou, PR China; d Metabolic Disease Research, Department of Cardiovascular Sciences, Temple University Lewis Katz School of Medicine, Philadelphia, PA, USA; e School of Biomedical Engineering, Shenzhen Campus of Sun Yat-sen University, Shenzhen, 518107, Guangdong, PR China

**Keywords:** Statins, Sterol regulatory element-binding protein 1, Type 2 diabetes mellitus, Myocardial lipid peroxidation

## Abstract

Statins therapy is efficacious in diminishing the risk of major cardiovascular events in diabetic patients. However, our research has uncovered a correlation between the prolonged administration of statins and an elevated risk of myocardial dysfunction in patients with type II diabetes mellitus (TIIDM). Here, we report the induction of sterol regulatory element-binding protein 1 (SREBP1) activation, associated lipid peroxidation, and the consequent diabetic myocardial dysfunction after statin treatment and explored the underlying mechanisms. In *db/db* mice, we observed that 40 weeks atorvastatin (5 and 10 mg/kg) and rosuvastatin (20 mg/kg) administration exacerbated diabetic myocardial dysfunction by echocardiography and cardiomyocyte contractility assay, increased myocardial inflammation and fibrosis as shown by CD68, IL-1β, Masson's staining and Collagen1A1 immunohistochemistry (IHC) staining, increased respiratory exchange ratio (RER) by metabolic cage system assessment, exacerbated mitochondrial structural pathological changes by transmission electron microscopy (TEM) examination, increased deposition of lipid and glycogen by TEM, Oil-red and periodic acid-schiff stain (PAS) staining, which were corresponded with augmented levels of myocardial SREBP1 protein and lipid peroxidation marked by 4-hydroxynonenal (4-HNE) staining. Comparable myocardial fibrosis was also observed in KK-ay and low-dose streptozotocin (STZ)-induced TIIDM mice. Elevated SREBP1 levels were observed in the heart tissues from diabetic patients, which was positively correlated with their myocardial dysfunction. To elucidate the role of statin induced SREBP1 in lipid peroxidation and lipid deposition and related mechanism, we cultured neonatal mouse primary cardiomyocytes (NMPCs) and treated them with atorvastatin (10 μM, 24 h), tracing with [U–^13^C]-glucose and evaluating for SREBP1 expression and localization. We found that statin treatment elevated de novo lipogenesis (DNL) and the levels of SREBP1 cleavage-activating protein (SCAP), reduced the interaction of SCAP with insulin-induced gene 1 (Insig1), and enhance SCAP/SREBP1 translocation to the Golgi, which facilitate SREBP1 cleavage leading to its nuclear *trans*-localization and activation in NMPCs. Ultimately, SREBP1 knockdown or l-carnitine mitigated long-term statins therapy induced lipid peroxidation and myocardial fibrosis in low-dose STZ treated *SREBP1*^*+/−*^ mice and l-carnitine treated *db/db* mice. In conclusion, we demonstrated that statin therapy may augment DNL by activating SREBP1, resulting in myocardial lipid peroxidation and lipid deposition.

## Highlights

• SREBP1 activation drives myocardial lipid peroxidation and deposition in diabetic myocardial dysfunction.
• Long-term statins treatment induces myocardial dysfunction, inflammation and fibrosis.
• Statin-induced myocardial lipid peroxidation and deposition link to SREBP1-dependent lipogenesis in TIIDM.
• Statins and l-carnitine combined therapy effectively mitigates statin-induced myocardial lipid peroxidation.

## 1 Introduction

Diabetic myocardial dysfunction is a major contributor of mortality among individuals with type II diabetes mellitus (TIIDM) [1]. Hyperlipidemia, particularly elevated levels of low-density lipoprotein cholesterol (LDL-C), is a recognized risk factor for cardiovascular diseases (CVDs) in TIIDM patients [1]. Meta-analyses have shown a proportional reduction in all-cause mortality per mmol/L reduction in LDL-C in TIIDM patients [2], leading to the strong recommendation of lipid-lowering therapies, especially statins, which are recommended as first-line treatments in clinical guidelines [3]. Statins, by inhibiting HMG-CoA reductase, effectively reduce LDL-C levels and significantly decrease the risk of major cardiovascular events [[4], [5], [6]].

Although statins are frequently prescribed for long-term management for hyperlipidemia, there is limited research on their potential adverse effects on organ damage, particularly in diabetic conditions. However, the debate about the symptomatic adverse effects of statins has not been resolved. The most commonly reported adverse effect is statin-associated muscle symptoms (SAMS), present in 10–29 % of patients taking statin therapy according to observational studies [7]. Besides muscles, our previous research has indicated that long-term statins administration exacerbated diabetic nephropathy by increasing insulin resistance (IR) in diabetic mice [8]. However, the prevailing academic consensus is that long-term statins therapy results in few symptomatic adverse events, significantly fewer than the clinical benefits [9,10], and had not attracted enough attention. Balancing the benefits and adverse events of statins is a critical clinical consideration for all cardiovascular physicians, particularly in the treatment of diabetic patients. Moreover, most clinical studies have concentrated on short-term statin administration, and the impact of long-term use on the severity of statin-related adverse events remains unclear.

Understanding the precise mechanisms that underlie cardiac metabolic disorders in diabetes and developing novel therapeutic strategies to mitigate the adverse events of statin is crucial. SREBPs are transcription factors pivotal in maintaining lipid homeostasis, responsible for activating genes linked to triglyceride, fatty acid, and cholesterol synthesis [11]. The induction of SREBP1 has been associated with lipid-mediated peroxidation, contributing to various metabolic diseases, including obesity, type II diabetes mellitus (TIIDM), hyperlipidemia, hepatosteatosis, and atherosclerosis [12]. Our previous research has indicated that SREBP1 mediates lipid peroxidation and lipid deposition in diabetic kidneys [8]. Nonetheless, its regulatory function in myocardial lipid peroxidation during the initiation and progression of diabetic myocardial dysfunction remains unclear. In this study, we demonstrate that myocardial SREBP1 acts as a central regulator in de novo lipogenesis (DNL), myocardial lipid peroxidation and lipid deposition, in response to progression of diabetic myocardial dysfunction. Statin therapy may exacerbate DNL by activating SREBP1, thereby promoting myocardial lipid deposition.

In this study, we employed human cardiac tissue samples, genetically engineered mouse models, and *in vitro* experiments to examine the myocardial adverse events of long-term statins therapy in TIIDM. We aimed to investigate whether SREBP1 induction mediates long-term statins therapy related myocardial lipid peroxidation and lipid deposition in TIIDM, thereby elucidating the mechanisms behind these phenomena. Our findings indicate that the induction and activation of SREBP1 mediate long-term statins therapy-related myocardial DNL, lipid peroxidation, and lipid deposition. These results contribute to a more profound comprehension of the role of cardiac lipid metabolism in the treatment of diabetic hyperlipidemia.

## 2 Results

### 2.1 Elevated blood lipids, myocardial lipid deposition, and myocardial SREBP1 upregulation in TIIDM mice

**Fig. 1:**
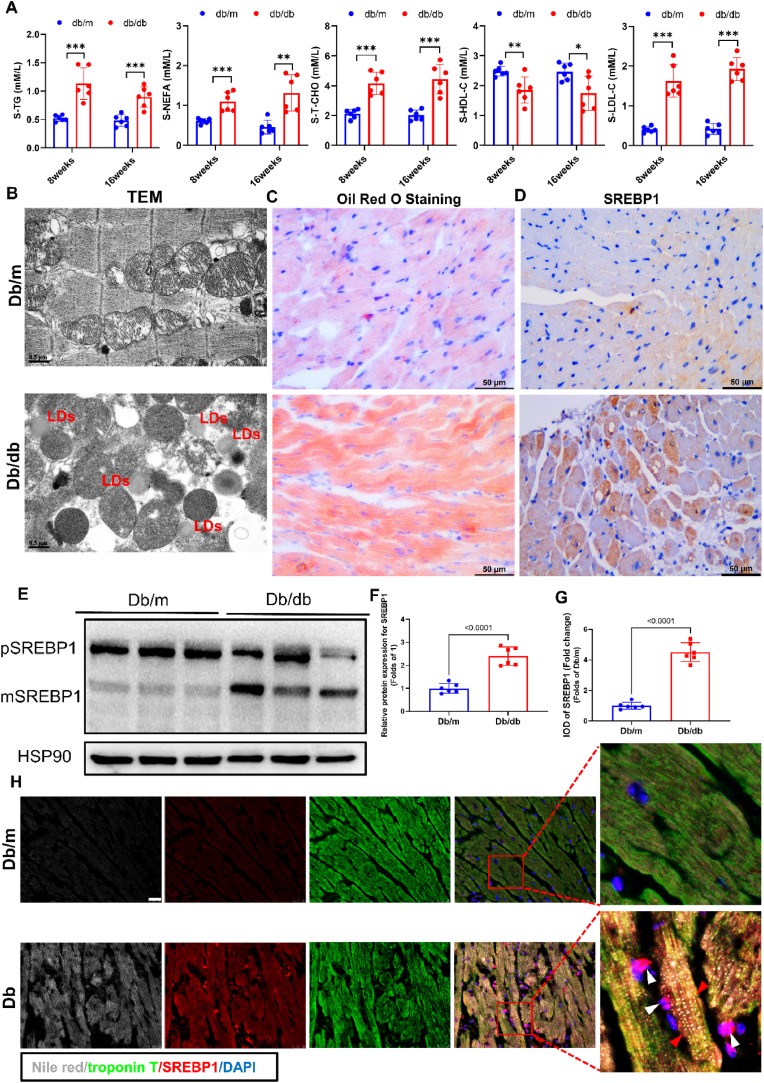
Elevated blood lipids, myocardial lipid deposition, and myocardial SREBP1 upregulation in TIIDM Mice. **(A)** Serum lipid profile in *db/db* mice at 8 week and 16 weeks. *n* = 6 in each group. **(B)** Representative TEM images in the myocardium. Original magnification × 26,500, scale bar = 0.5 μm. **(C)** Representative Oil-Red O staining images in the myocardium. **(D)** Immunohistochemistry staining of SREBP1 in the heart from each group. Original magnification × 400, scale bar = 50 μm. **(*E***–**F)** Representative immunoblot images of SREBP1 in mouse heart lysates and quantification. *n* = 6 in each group. **(G)** Analysis of SREBP1 protein expression in the myocardium according to immunohistochemical staining. *n* = 6 in each group. **(H)** Immunofluorescence staining of protein SREBP1 (red), Nile red (gray), and cardiomyocyte marker Troponin T (green) in the myocardium. Red arrows indicate myocardial lipid droplets; white arrows indicate SREBP1 is transferred into the nucleus. Original magnification × 1000, scale bar = 20 μm. All results were presented as the means ± SEM. Student's t-test was used for statistical analysis. (For interpretation of the references to colour in this figure legend, the reader is referred to the Web version of this article.)

In our study, *db/db* mice exhibited severe hyperlipidemia in the early stages (8 weeks and 16 weeks) of diabetes, characterized by a significant rise in triglyceride (TG), nonesterified fatty acid (NEFA), and total cholesterol (TCHO) levels, along with a marked increase in low-density lipoprotein cholesterol (LDL-C) and a corresponding decrease in high-density lipoprotein cholesterol (HDL-C) at 8 and 16 weeks (Fig. 1A). Diabetic dyslipidemia-induced cardiac lipid peroxidation and deposition are recognized as pivotal contributors to myocardial dysfunction. Accordingly, we assessed lipid accumulation within cardiomyocytes using transmission electron microscopy (TEM) and Oil Red O staining, which demonstrated substantial lipid droplet aggregation in the cardiac tissue of *db/db* mice (Fig. 1B–C). We hypothesized a correlation between lipid deposition in the diabetic myocardium and the activity of SREBP1, a key regulator of lipid metabolism. Through immunohistochemistry and immunoblotting, we determined that SREBP1 expression in the myocardium was significantly elevated in *db/db* mice (Fig. 1D–G). Further investigation with Nile Red and SREBP1 immunofluorescence staining confirmed a positive association between heightened SREBP1 expression and lipid droplet deposition in the hearts of *db/db* mice (Fig. 1H). In our study of diabetic myocardial tissue, we observed a positive correlation between the accumulation of lipids and the upregulation of SREBP1, along with its subsequent translocation into the nucleus. In summary, our findings indicate that increased SREBP1 expression in the diabetic myocardium is linked to enhanced lipid deposition.

### 2.2 SREBP1 is elevated in cardiac tissues of diabetic patients

**Fig. 2:**
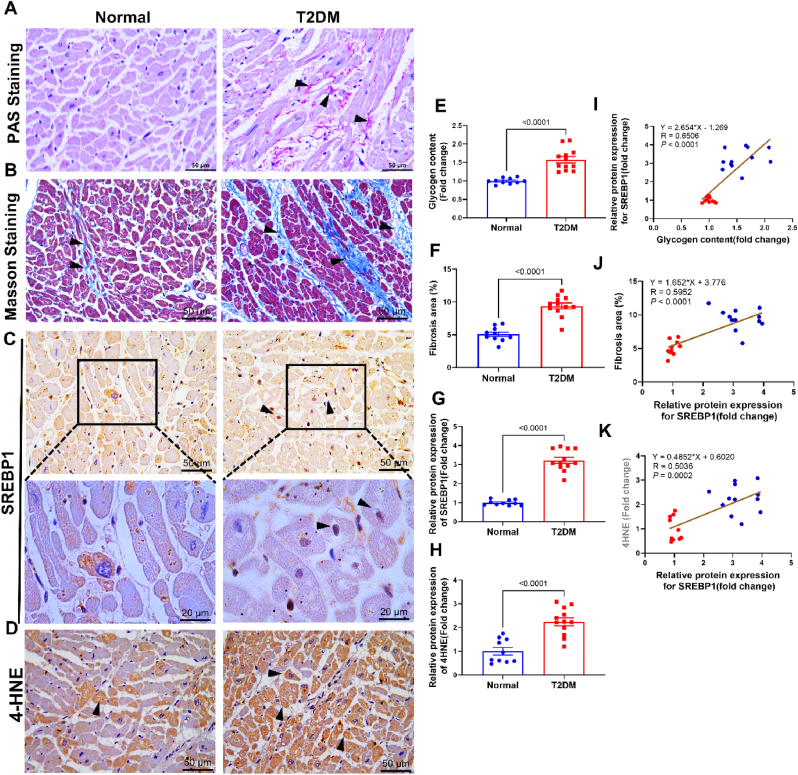
SREBP1 is elevated in cardiac tissues of diabetic patients. **(A**–**D)** Representative Periodic acid–Schiff (PAS) staining, Masson's trichrome staining, SREBP1 immunohistochemical staining, and 4-HNE immunohistochemical staining images in the myocardium from normal and TIIDM patients. Black arrows indicate glycogen deposition (A), collagen deposition (B), SREBP1 nuclear translocation (C), and 4-HNE positive expression (D) in the myocardium. Original magnification × 400, scale bar = 50 μm. **(*E***–**H)** Quantitative analysis of glycogen, fibrosis, SREBP1 expression, and 4-HNE expression within the myocardium. **(I–K)** Linear correlation analysis. Red dots indicate normal individuals and blue dots indicate TIIDM patients. *n* = 10 in the Normal group and *n* = 12 in the TIIDM group. Data are expressed as means ± SEM. A two-tailed unpaired Student's t-test was used for analysis in (E–H). For correlation analysis, linear regression models were performed and the goodness of fit for regression models was assessed using R values. (For interpretation of the references to colour in this figure legend, the reader is referred to the Web version of this article.)

Subsequently, we assessed the expression of SREBP1 in human cardiac tissue samples. Heart specimens from two categories of decedents (Normal and TIIDM patients), ensuring age and sex matching (refer to Supplementary Table 1). Hearts from TIIDM patients exhibited pronounced glycogen accumulation, myocardial fibrosis, and elevated levels of SREBP1 and 4-hydroxynonenal (4-HNE), a biomarker for oxidative/nitrosative stress and lipid peroxidation, in contrast to the control group (as shown in Fig. 2A–H). A linear regression analysis conducted on 22 cardiac samples disclosed a significant positive correlation between glycogen content and SREBP1 levels (Fig. 2I), as well as between SREBP1 levels and both the severity of myocardial fibrosis (Fig. 2J) and 4-HNE expression (Fig. 2K). These results imply that SREBP1 induction may be correlated with lipid peroxidation, glycogen deposition, and fibrosis in diabetic heart.

### 2.3 Long-term statins treatment exacerbated myocardial dysfunction and elevated myocardial lipid peroxidation, inflammation and fibrosis in TIIDM mice

**Fig. 3:**
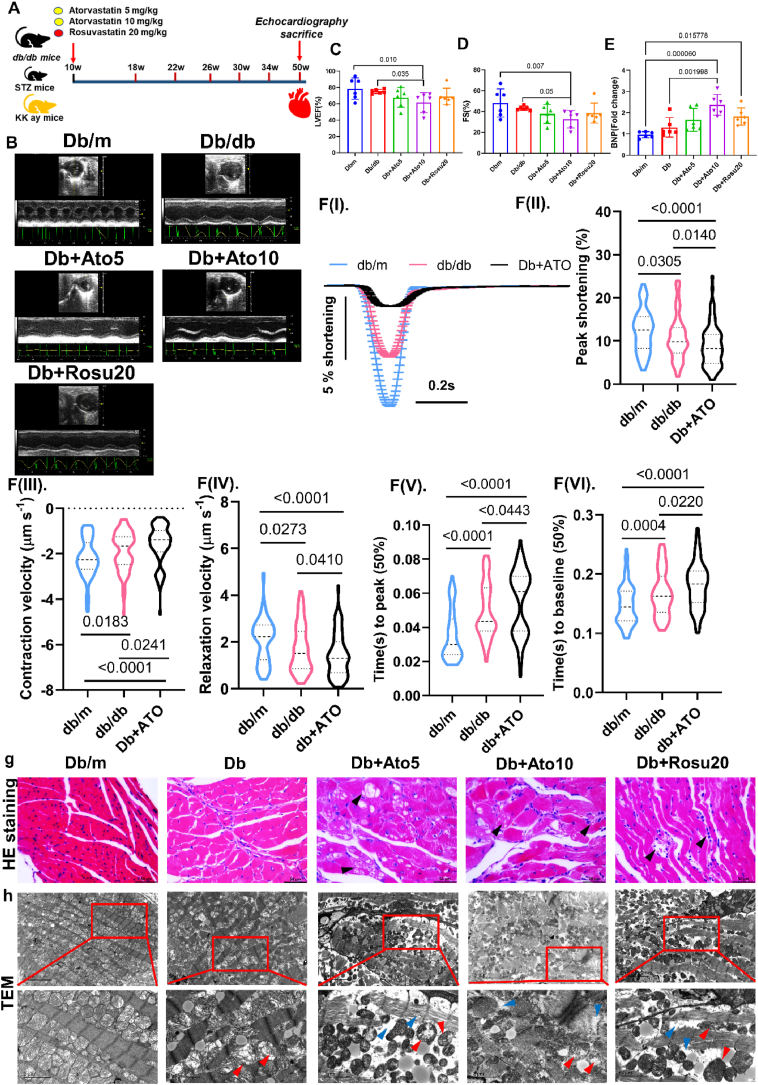
Long-term statins treatment exacerbated myocardial dysfunction and elevated myocardial lipid peroxidation, inflammation and fibrosis in TIIDM mice. **(A)** Timeline of experimental design. Investigation of the effect of long-term administration of statins on cardiac function using the model of *db/db* mice, STZ mice, and KK-ay mice. In brief, three types of TIIDM mice were administered Atorvastatin at dosages of 5 or 10 mg/kg and Rosuvastatin at a dosage of 20 mg/kg daily by gavage for 40 weeks, beginning after a stable increase in blood glucose levels was observed at the start of the 10th week. Echocardiography was performed at the indicated time points.**(B)** Representative left ventricular M-mode echocardiographic tracings from each group of *db/db* mice. **(C**–**D)** Quantification of ejection fraction and fractional shortening. *n* = 6 in each group. **(E)** Detection of BNP in serum in statin-treated *db/db* mice. *n* = 6 in each group. **(F)** The effect of long-term administration of statins on cardiomyocyte contractility assay in *db/db* mice. **(F–I)** Representative sarcomere shortening. **(F-II)** Peak shortening. **(F-III)** Contraction velocity. **(F-IV)** Relaxation velocity. **(F–V)** Time(s) to peak (50 %). **(F-VI)** Time(s) to baseline (50 %). Violin plots show lines at the median (solid) and quartiles (dashed). Two-tailed unpaired *t*-test (A–H). *n* = 3 in each group. p-values are indicated. **(G)** Representative HE staining images in the myocardium. Black arrows indicate vacuolar degeneration and inflammatory infiltrate of cardiomyocytes. Original magnification × 400, scale bar = 50 μm. **(H)** Representative TEM images in the myocardium. Red arrows indicate cardiac mitochondrial swelling and condensation; blue arrows indicate myofibril breakage, lysis, and necrosis. Original magnification × 8000 or × 20,000, scale bar = 5 or 2 μm. Data are expressed as means ± SEM. One-way ANOVA with Tukey post hoc test was used for statistical analysis. (For interpretation of the references to colour in this figure legend, the reader is referred to the Web version of this article.)

Statins are frequently utilized as primary lipid-lowering agents in clinical settings to manage hyperlipidemia and prevent lipid peroxidation in target organs. Echocardiographic assessments revealed a significant reduction in both ejection fraction and fractional shortening in the groups treated with statins compared to the Db group (Fig. 3B–D, see Supplementary Table 1 for details). Additionally, serum brain natriuretic peptide (BNP) levels were markedly elevated post-treatment with statins (Fig. 3E), suggesting exacerbation of myocardial dysfunction in TIIDM mice [13]. Single-cell contraction assays were conducted on freshly isolated primary cardiomyocytes from mice across different groups (Db/m, Db, and Db + ATO) using an electrical stimulator (Fig. 3F–I). The sarcomere shortening trace and sarcomere peak shortening of isolated cardiomyocytes showed a similar trend to the ejection fraction in vivo, indicating that the contraction of isolated cardiomyocytes measured ex vivo corresponds to the ejection fraction in vivo (Fig. 3F–II). Notably, Db + ATO group cardiomyocytes exhibited lower levels of sarcomere shortening, decreased contraction velocity by 15.8 %, and relaxation velocity by 17.6 % compared to *db/db* cardiomyocytes (Fig. 3F–III to IV). Histopathologic examination indicated cardiomyocyte degeneration and inflammatory infiltrates in *db/db* mice, especially in statin-treated mice (Fig. 3G). TEM analysis showed typical myofibrillar arrangement in control mice but significant degeneration in statin-treated *db/db* mice (Fig. 3H). Furthermore, statin-treated *db/db* mice exhibited mitochondrial swelling and disarray, along with significant accumulations (Fig. 3F–V to VI).

Upon further analysis, no significant pathological alterations were detected in the hearts of *db/m* mice following long-term statins administration (Supplementary Figs. 1A–C). Comparable cardiac pathological changes to those observed in db/db mice were identified in both KK-ay mice and low-dose streptozotocin (STZ)-induced TIIDM mice. Notably, Masson's staining indicated that long-term statins administration significantly exacerbated myocardial fibrosis in the heart of KK-ay mice and low dose STZ-induced TIIDM mice (Supplementary Figs. 2A–D). These findings imply that long-term statins administration may exacerbate myocardial dysfunction in TIIDM mice, with no observed adverse effects in *db/m* mice.

Statin-treated *db/db* mice demonstrated myocardial functional impairment, including progressive reductions in relaxation and contractility as assessed by echocardiography and single-cell contraction assays. Additionally, an increase in CD68-positive macrophage infiltration and IL-1β expression was observed in statin-treated *db/db* mice (Supplementary Figs. 3A–B/G). Statins treatment significantly enhanced myocardial lipid peroxidation, evidenced by an elevated level of 4-Hydroxynonenal (4-HNE) (Supplementary Fig. 3C), and fibrosis, indicated by an expanded fibrotic area (Supplementary Fig. 3D-E, H-J). Collectively, these findings indicate that long-term statins treatment induces cardiac structural changes in *db/db* mice.

### 2.4 Upregulation of cardiac SREBP1 lipogenesis in statin-treated *db/db* mice

**Fig. 4:**
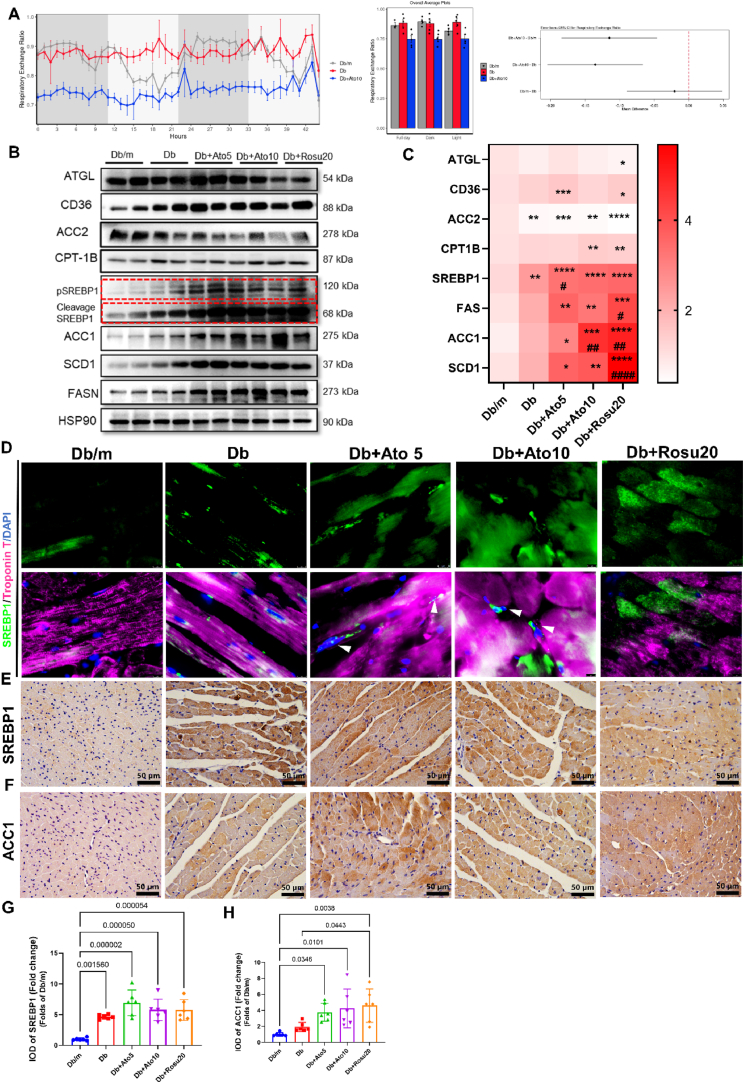
Upregulation of cardiac SREBP1 lipogenesis in statin-treated *db/db* Mice. **(A)** Respiratory exchange ratio (RER) of *db/db* mice after statin treatment at the end of the study. *n* = 5 in each group. **(B–C)** Representative immunoblot images and quantification of ATGL, CD36, ACC2, SREBP1, ACC1, SCD1, and FASN in the heart tissues. HSP90 was used as an internal control. *n* = 6 in each group. **(D)** Immunofluorescence staining of protein SREBP1 (green) and cardiomyocyte marker Troponin T (pink) in the myocardium. White arrows indicate SREBP1 nuclear translocation. Original magnification × 1000, scale bar = 20 μm. **(*E***–**F)** Immunohistochemistry staining of SREBP1 and ACC1 in the heart from each group of *db/db* mice. Original magnification × 400, scale bar = 50 μm. **(G**–**H)** Analysis of SREBP1 and ACC1 protein expression in the myocardium according to immunohistochemical staining. *n* = 6 in each group. Data are expressed as means ± SEM. One-way ANOVA with Tukey post hoc test was used for statistical analysis. (For interpretation of the references to colour in this figure legend, the reader is referred to the Web version of this article.)

We hypothesized that statins modify cardiac metabolic effects in *db/db* mice. Indirect calorimetry revealed a significant reduction in the respiratory exchange ratio (RER) among statin-treated *db/db* mice, indicating a shift in energy source from carbohydrates to lipids (Fig. 4A). The energy expenditure, energy balance, food consumed, and water consumed also showed significant differences (Supplementary Figs. 4A–G), implying that statins could influence cardiac lipid metabolism. Examination of genes related to fatty acid (FA) metabolism indicated no significant alterations in those involved in lipolysis (Adipose triglyceride lipase, ATGL), FA uptake (Cluster of differentiation 36, CD36), and FA oxidation (Acetyl-CoA Carboxylase 2, ACC2; Carnitine Palmitoyltransferase 1 B, CPT1B) (Fig. 4B–C). However, the SREBP1 lipogenesis pathway, including SREBP1, Acetyl Coenzyme A Carboxylase 1 (ACC1), Fatty Acid Synthase (FASN), and Stearoyl-CoA Desaturase 1 (SCD1), was markedly upregulated (Fig. 4B–C). Immunoblotting and immunofluorescence showed significant upregulation and nuclear translocation of SREBP1-N in statin-treated *db/db* mice myocardium (Fig. 4D). SREBP1 and downstream targeting enzymes such as ACC1 were also significantly upregulated in statin-treated *db/db* mice myocardium, shown by immunohistochemical staining (Fig. 4*E*–H). These results suggest that statins may induce myocardial dysfunction via the SREBP1 lipogenesis pathway.

### 2.5 Amelioration of statin-exacerbated myocardial lipid deposition in TIIDM mice by SREBP1 genetic knockdown

**Fig. 5:**
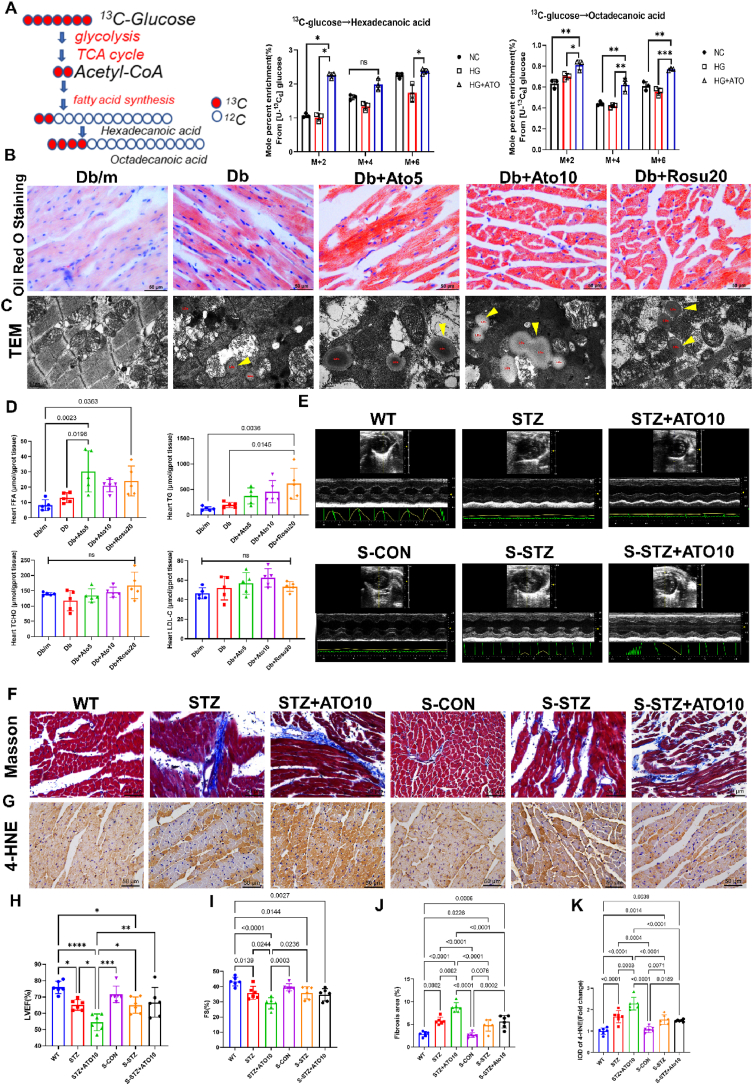
Amelioration of statin-exacerbated myocardial lipid deposition in TIIDM mice by SREBP1 genetic knockdown. **(A)** Metabolic flux analysis of atorvastatin-treated NMPCs labeled by [U–^13^C] glucose, utilized for de novo fatty acid synthesis (left panel). The incorporation of ^13^C into Hexadecanoic acid and Octadecanoic acid are shown in the middle and right panels. NMPCs were subjected to starvation in a serum-free medium overnight, then divide into 3 groups, respectively cultured in [U–^13^C]-glucose 1.0 mmol/L (group NG), cultured in a [U–^13^C]-glucose 4.5 mmol/L (group HG), the group HG + ATO treated with [U–^13^C]-glucose 4.5 mmoL/l and atorvastatin 10 μmol/L (Sigma-Aldrich, PHR1422). All groups NMPCs were cultured for 24 h. The experiment was repeated three times. **(B)** Representative Oil-Red O staining images in the myocardium from each group of *db/db* mice. **(C)** Representative TEM images of left ventricular walls of the hearts from each group of *db/db* mice. LDs indicate lipid droplets. Yellow arrows represent lipid droplets deposited in cardiomyocytes. Scale bar = 0.5 μm. **(D)** Determination of cardiac tissue TCHO, LDL-C, TG, and FFA contents from each group of *db/db* mice. *n* = 5 in each group. **(E)** Representative left ventricular M-mode echocardiographic tracings from each group of low doses STZ-induced TIIDM mice. *S*–CON indicates SREBP1-deficient mice. *S*-STZ indicates low doses STZ-induced TIIDM mice of SREBP1-deficient mice. *S*-STZ + ATO10 indicates low doses STZ-induced TIIDM mice of SREBP1-deficient mice treated with atorvastatin for 30 weeks. **(F**–**G)** Representative Masson's trichrome staining and 4-HNE (IHC) images of left ventricular walls of the hearts from each group. Original magnification × 400, scale bar = 50 μm. **(H–I)** Quantification of ejection fraction and fractional shortening from each group. *n* = 6 in each group. **(J)** Semi-quantification analysis of fibrotic areas in the myocardium from each group. *n* = 6 in each group. **(K)** Semi-quantification analysis of 4-HNE expression from each group. *n* = 6 in each group. Data are expressed as means ± SEM. One-way ANOVA with Tukey post hoc test was used for statistical analysis. (For interpretation of the references to colour in this figure legend, the reader is referred to the Web version of this article.)

The relationship between elevated SREBP1 expression in cardiomyocytes and enhanced de novo lipogenesis (DNL) remains unclear. Utilizing [U–^13^C]-glucose metabolic flux analysis, we investigated the impact of atorvastatin on FA synthesis in neonatal mouse primary cardiomyocytes (NMPCs) under conditions of high glucose. Our findings confirmed that atorvastatin treatment significantly increased the synthesis of hexadecanoic and octadecanoic acids in NMPCs under high-glucose conditions (Fig. 5A). Subsequently, we examined the in vivo effects of statins on cardiac lipid deposition. Both Oil Red O staining and TEM revealed that statins treatment led to substantial lipid droplet accumulation in cardiomyocytes (Fig. 5B–C). Concurrently, we measured lipid levels in the myocardial tissue of *db/db* mice and observed that statin treatment notably elevated cardiac free fatty acids (FFAs) and TG levels, whereas TCHO and LDL-C levels remained unchanged (Fig. 5D). The presence of aberrant SREBP1 overexpression and lipid deposition was further corroborated by Nile Red staining and immunofluorescence (Supplementary Fig. 5A). Collectively, these observations preliminarily suggest that long-term statins therapy could enhance DNL in cardiomyocytes by aberrantly activating the SREBP1 pathway, potentially leading to myocardial lipid deposition.

To test our hypothesis, we generated SREBP1-deficient mice and induced TIIDM by administering a low dose of STZ. We assessed the expression of SREBP1 in the cardiac tissue of these low-dose STZ-induced SREBP1-deficient TIIDM mice by immunohistochemistry and quantified the expression levels of lipid metabolism-related genes via immunoblotting (Supplementary Figs. 6A–C). Our results revealed a significant reduction in SREBP1 expression within the heart of low-dose STZ-induced SREBP1-deficient TIIDM mice. Additionally, the expression levels of SREBP1 target genes, including ACC1, FASN, and SCD1, were markedly reduced, confirming the successful generation of SREBP1-deficient mice. We further examined the body weight, fasting blood glucose levels and glucose tolerance test (GTT) to assess the baseline glycemic status. It can be seen that SREBP1-deficiency does not affect the body weight and blood glucose level of TIIDM mice (Supplemental figure 6D-E). However, the AUC of GTT in low dose STZ-induced SREBP1-deficient TIIDM mice was significantly reduced, indicating that SREBP1-deficient exhibit an enhanced capability for glucose homeostasis (Supplemental figure 6F-G). In low-dose STZ-induced SREBP1-deficient TIIDM mice, long-term statin therapy (*S*-STZ + ATO group) significantly improved cardiac function, as demonstrated by increased ejection fraction and fractional shortening, and reduced cardiac fibrosis and 4-HNE levels when compared to STZ + ATO group (Fig. 5*E*–K). These findings suggest that myocardial lipid peroxidation is associated with the elevated expression of SREBP1, and genetic reduction of SREBP1 mitigated statin-induced cardiac fibrosis and dysfunction.

### 2.6 Glucose-mediated glycosylation promotes SREBP-cleavage activating protein (SCAP) trafficking to the Golgi, leading to SREBP activation in NMPCs

**Fig. 6:**
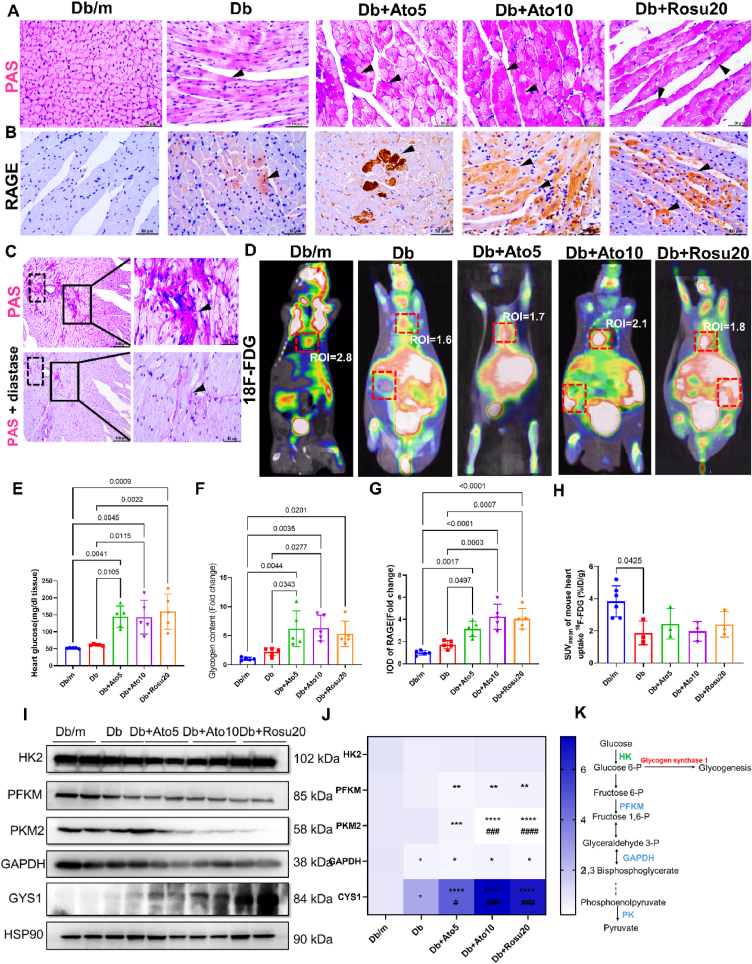
Long-term statins administration increases myocardial glucose accumulation in *db/db* mice via enhanced glycogenesis. **(A**–**B)** Representative PAS staining and the receptor of advanced glycation endproducts (RAGE) immunohistochemical staining images of left ventricular walls of the hearts from each group of *db/db* mice. Black arrows indicate glycogen deposition (A) and RAGE positive expression (B). **(C)** Heart sections treated with diastase as a negative control for PAS staining. Original magnification × 400, scale bar = 50 μm. **(D)** Cardiac glucose uptake was evaluated with ^18^F-fluorodeoxyglucose (18 F-FDG). Representative images of ^18^F-FDG positron emission tomography/computed tomography (PET/CT) from each group of *db/db* mice. The mean value standard uptake value (SUV_mean_) based on region of interest (ROI) analysis was quantified via PET/CT. **(E)** Determination of cardiac tissue glucose contents from each group of *db/db* mice. *n* = 5 in each group. **(F)** Analysis of glycogen content in the heart according to PAS staining. *n* = 5 in each group. **(G)** Analysis of RAGE protein expression in the myocardium according to immunohistochemical staining. *n* = 5 in each group. **(H)** SUV_mean_ of mouse heart uptake of ^18^F-FDG. *n* = 3 in each group. **(I)** Representative immunoblot images of HK2, PFKM, PKM2, GAPDH, and GYS1 in the heart tissues. HSP90 was used as an internal control. **(J)** Quantification of HK2, PFKM, PKM2, GAPDH, and GYS1 protein expression in the myocardium according to immunoblot. *n* = 6 in each group. **(K)** Schematic diagram showing the changes in glycolysis-related enzymes following long-term administration of statins in *db/db* mice. Data are expressed as means ± SEM. One-way ANOVA with Tukey post hoc test was used for statistical analysis.

We examined the mechanisms underlying statin-induced upregulation of SREBP1 expression and its nuclear translocation. Periodic acid-Schiff (PAS) staining and glucose content measurement revealed substantial glycogen accumulation in the cardiomyocytes of statin-treated *db/db* mice (Fig. 6A/C/E/F). PAS staining also indicated significant glycogen deposition in the kidneys and skeletal muscle of *db/db* mice following statin treatment, suggesting that long-term statin therapy leads to glycogen deposition in peripheral organs, not limited to the myocardium (Supplementary Fig. 7A and B). Furthermore, statins treatment significantly upregulated the expression of the receptor for advanced glycation end-products (RAGE) in cardiomyocytes, potentially perpetuating oxidative stress (Fig. 6 B/G, Supplementary Figs. 7C–D). To elucidate why statins induce glycogen deposition in cardiomyocytes, we utilized ^18^F-FDG PET/CT imaging, which showed no difference in cardiac glucose uptake between statin-treated and control *db/db* mice (Fig. 6D/H). Western blot analysis revealed decreased expression of glycolytic enzymes, including Hexokinase 2 (HK2), Phosphofructokinase (PFKM), pyruvate kinase M2 (PKM2), and glyceraldehyde-3-phosphate dehydrogenase (GAPDH), alongside increased expression of glycogen synthase 1 (GYS1) in the statin-treated group (Fig. 6I–K). This indicates that glycogen deposition in cardiomyocytes following long-term statin therapy may result from enhanced glycogen synthesis rather than increased glucose uptake. We further investigated the molecular basis for increased glycogen synthesis in cardiomyocytes, discovering that the protein kinase B (AKT)/mammalian target of rapamycin (mTOR) signaling pathway, crucial for glucose metabolism, was significantly activated, as evidenced by upregulated phosphorylated AKT and phosphorylated p70S6 kinase (Supplementary Figs. 8A–C). Therefore, we believed that these effects are at least partially mediated by increased IR, confirmed by activation of the AKT-mTOR signaling pathway. In summary, our data confirm that long-term statins therapy may enhance glycogen deposition in cardiomyocytes by activating the AKT-mTOR signaling pathway, thereby increasing glycogen synthesis.

**Fig. 7:**
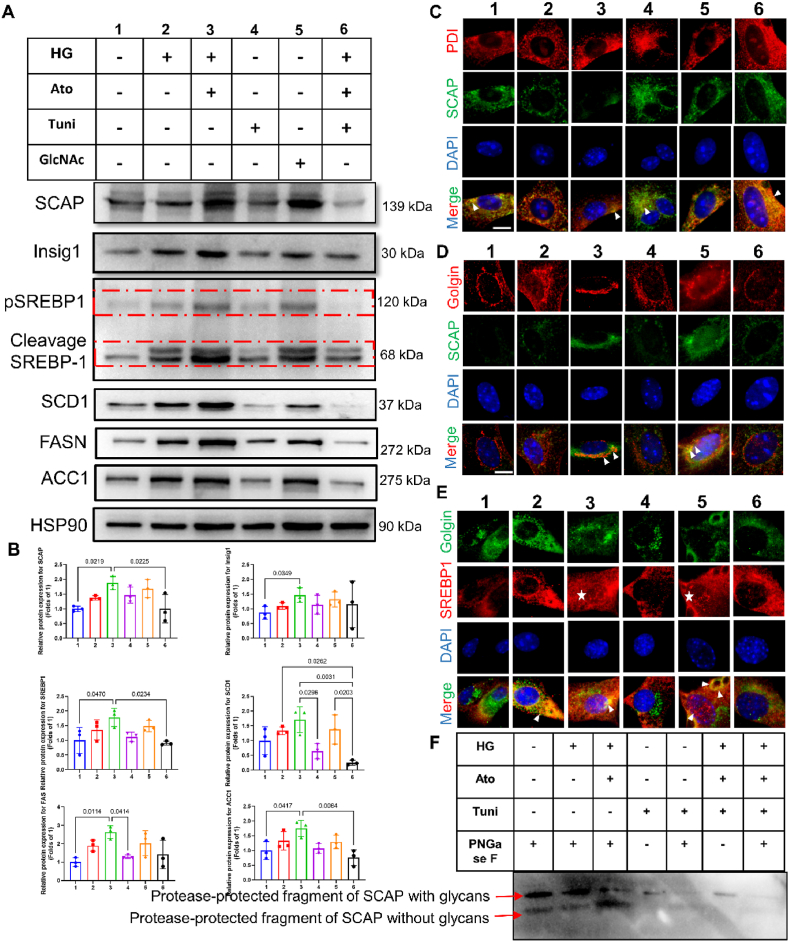
Statin enhance intracellular glucose accumulation, promoting SCAP N-glycosylation and trafficking to the Golgi, leading to SREBP1 nuclear translocation. NMPCs were subjected to starvation in a serum-free medium overnight, then divided into six groups: group 1 cultured in glucose 1.0 mmol/L, group 2 cultured in glucose 4.5 mmol/L, group 3 treated with glucose 4.5 mmol/L and atorvastatin 10 mmol/L, group 4 treated with tunicamycin (1 μg/mL), group 5 treated with GlcNAc (20 mM), and group 6 treated with glucose 4.5 mmol/L, atorvastatin 10 mmol/L, and tunicamycin (1 μg/mL). All groups of NMPCs were cultured for 24 h **(A**–**B)**. Representative immunoblot images and quantification of SCAP, INSIG1, SREBP1, SCD1, FASN, and ACC1. HSP90 was used as an internal control. The demonstrated bands were typical from three experimental repeats. **(C)** Immunofluorescence images of SCAP (green) sub-cellular localization in relation to the endoplasmic reticulum protein marker PDI (red) and nuclear DAPI staining (blue) in NMPCs with the same treatment procedure as panel A. White arrows indicate merged SCAP and PDI signals (yellow). **(D)** Immunofluorescence images of SCAP (green) sub-cellular localization in relation to the Golgi protein marker Golgin (red) and nuclear DAPI staining (blue) in NMPCs with the same treatment procedure as panel A. White arrows indicate merged SCAP and Golgin signals (yellow), indicating translocation of endogenous SCAP into the Golgi. **(E)** Immunofluorescence images of SREBP1 (red) sub-cellular localization in relation to the Golgi protein marker Golgin (green) and nuclear DAPI staining (blue) in NMPCs with the same treatment procedure as panel A. White stars indicate translocation of endogenous SREBP1 into the nucleus; white arrows indicate merged SREBP1 and Golgin signals (yellow). Original magnification × 1000, scale bars = 20 μm. **(F)** N-glycans of SCAP were analyzed as described in the Supplementary materials and methods section. Data are expressed as means ± SEM. One-way ANOVA with Tukey post hoc test was used for statistical analysis. (For interpretation of the references to colour in this figure legend, the reader is referred to the Web version of this article.)

To explore the mechanisms by which glucose enhances SCAP protein levels and activates SREBP, NMPCs were cultured in the absence or presence of glucose (25 mmol/L) and atorvastatin (10 μmol/L) for 24 h, and SREBP1 and SCAP processing was analyzed by immunoblot and immunofluorescence microscopy. Additionally, N-acetylglucosamine (GlcNAc; 20 mmoL/l) facilitated N-glycosylation, and tunicamycin (Tuni; 1 mg/mL) was an effective inhibitor of N-glycosylation. The immunoblot data (Fig. 7 A/B) showed that GlcNAc enhanced SCAP protein levels and promoted SREBP-1 cleavage; conversely, exposure of cells to Tuni reduced both SCAP protein levels and SREBP-1 cleavage. As expected, exposure of cells to glucose with atorvastatin was as effective as treatment with GlcNAc at enhancing SCAP protein levels and promoting SREBP-1 cleavage, while adding Tuni at the same time inhibited these effects (Fig. 7A–B). In parallel, downstream targets for SREBP1, i.e., FASN, ACC1, SCD1, were consistent with SREBP1 (Fig. 7A–B). Immunofluorescence imaging showed that under Tuni exposure conditions, even with supplementation with high glucose and atorvastatin, SREBP1 was still retained in the endoplasmic reticulum (ER) membrane as shown by co-staining with protein disulfide isomerase (PDI), an ER membrane protein. Converse results were observed with GlcNAc or high glucose with atorvastatin treatment (Fig. 7 C). SREBP stability, transport to the Golgi and cleavage require the formation of a complex between SREBP and SCAP. We observed that GlcNAc or glucose with atorvastatin treatment had the same effect on SCAP and SREBP1 trafficking to the Golgi and subsequent SREBP1 activation, N-terminal fragment of epitope-tagged SREBP in the nuclear as shown by costaining with nuclear DAPI and Golgin, a Golgi protein marker (Fig. 7D–E). Opposite results were observed in the Tuni treatment.

Previous studies have confirmed that SCAP contains a luminal region (a.a. 540–707) with two N-glycosylation sites that are protected from proteolysis when intact membranes are treated with trypsin. This luminal fragment has a molecular weight of approximately 30 kDa and allows the resolution of individual glycosylation variants of SCAP by SDS-PAGE. Next, we wondered whether SCAP N-glycosylation is associated with atorvastatin treatment in human AC-16 cardiomyocytes. Within the physiological glucose concentration, N-glycans of SCAP showed that the apparent mass of the fewest trypsin protected fragments decreased (without glycans) after PNGase F digestion (Fig. 7 F, Lane 1). Excess glucose and atorvastatin showed that the apparent mass of the most trypsin protected fragments decreased (without glycans) after PNGase F digestion (Fig. 7 F, Lane 3). Tuni nearly abolished SCAP N- glycosylation, showing the two weaker bands of the most trypsin protected fragments, even in the presence of high glucose and atorvastatin stimulation. Our data demonstrated that under high-glucose conditions, atorvastatin induced SCAP N-glycosylation and promoted SCAP trafficking to the Golgi, leading to SREBP activation.

### 2.7 l-carnitine supplementation with statins therapy mitigated statin-induced myocardial dysfunction and lipid peroxidation

**Fig. 8:**
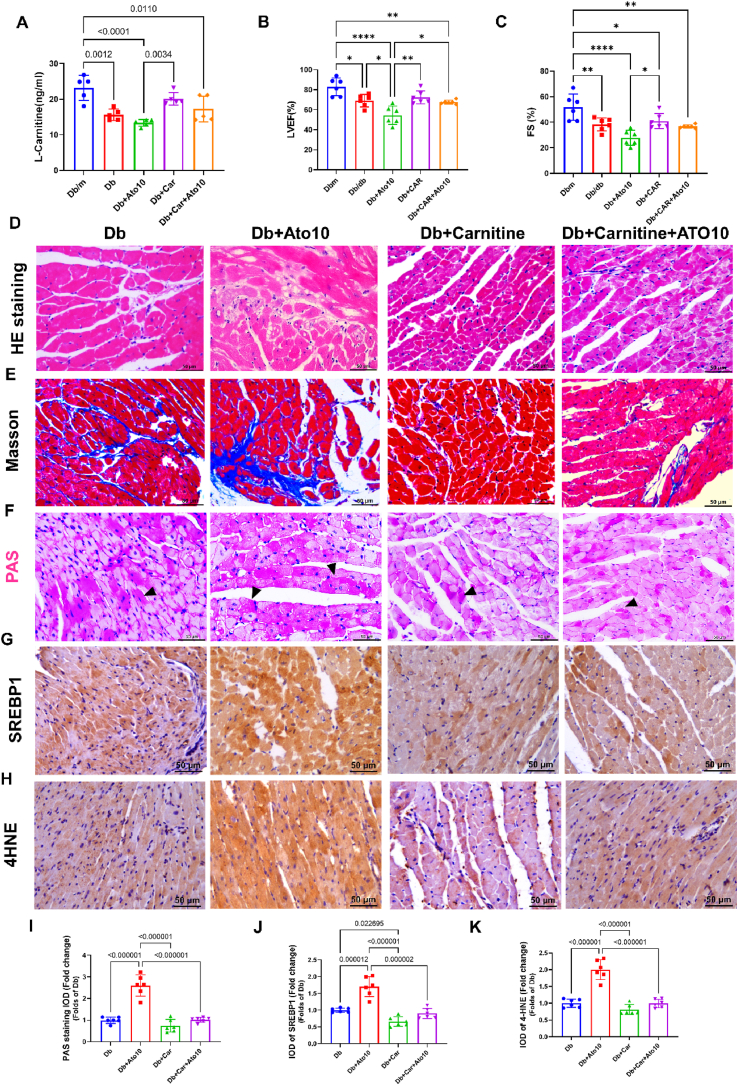
l-Carnitine supplementation with statins therapy mitigated statin-induced myocardial dysfunction and lipid peroxidation. Db/db mice were administered a combination of statin at a dosage of 10 mg/kg and l-carnitine at 300 mg/kg over a period of 40 weeks. Upon completion of the study, the hearts and plasma of the subjects were analyzed. Cardiac function was assessed using echocardiography. **(A)** Detection of l-carnitine content in heart tissue from each group. *n* = 5 in each group. **(B–C)** Quantification of ejection fraction and fractional shortening of left ventricular M-mode echocardiographic tracings. *n* = 6 in each group. **(D**–**H)** Representative HE staining, Masson's trichrome staining, PAS staining, SREBP1 staining, and 4-HNE staining images of heart sections. **(I–K)** Semi-quantification analysis of PAS staining, SREBP1 staining, and 4-HNE staining. *n* = 6 in each group. Data are expressed as means ± SEM. One-way ANOVA with Tukey post hoc test was used for statistical analysis.

We conducted further research to identify methods for alleviating myocardial dysfunction and lipid peroxidation associated with statins therapy in *db/db* mice. l-carnitine administration has been shown to enhance FAO and improve glucose metabolism, which may ameliorate statin-induced myocardial dysfunction. Initially, we observed that the l-carnitine levels in cardiac tissue of *db/db* mice were considerably lower compared to those in control mice (Fig. 8A). Supplementation of *db/db* mice with l-carnitine (300 mg/kg) notably elevated l-carnitine levels in heart tissue and counteracted the reduction in ejection fraction and fractional shortening typically associated with statin monotherapy (Fig. 8B–C). Histopathological examination revealed that l-carnitine supplementation diminished myocardial fibrosis and glycogen deposition (Fig. 8D–F). Most importantly, the combination of statins with l-carnitine significantly decreased cardiac SREBP1 expression and mitigated the extent of lipid peroxidation (Fig. 8G–H/J-K).

In conclusion, our findings indicate that long-term statins therapy stimulates myocardial DNL by augmenting intracellular glucose levels, facilitating SCAP N-glycosylation, and inducing the activation of SREBP1. This cascade of events contributes to myocardial lipid peroxidation and the subsequent development of cardiac dysfunction, as illustrated in Supplementary Fig. 9.

## 3 Discussion

Although statins have been widely as long-term therapy for hyperlipidemia, there is a relative scarcity of research on their potential adverse effects on organ systems, particularly in the context of diabetes. The majority of clinical studies have concentrated on the short-term effects of statins. The challenges associated with long-term statins administration and its characteristics remain understudied. This study applied a 40-weeks statin therapy in TIIDM mice and investigated its effects in myocardial pathology and underlying mechanism. Our study yielded three principal findings. First, long-term statins treatment induced myocardial dysfunction, inflammation and fibrosis, beside its benefit in reducing blood lipids in TIIDM. Second, statins treatment was observed to trigger myocardial lipid peroxidation and lipid deposition through SREBP1-activated DNL in TIIDM. Third, statins and l-carnitine combined therapy alleviated statin-induced myocardial lipid peroxidation. We concluded that SREBP1-activated lipogenesis is crucial for long-term statins treatment associated with myocardial lipid peroxidation and lipid deposition.

**Long-term statins treatment aggravated myocardial dysfunction, inflammation and fibrosis in TIIDM-**Our echocardiography and single-cell contraction assays indicated impaired cardiac contractile and relaxation, and slowed ventricular conduction velocity. Histopathologic analysis revealed significant myocardial interstitial fibrosis, inflammatory infiltrates and disrupted myofibrillar arrangement in statin-treated *db/db* mice. TEM examination demonstrated mitochondria swelling and cristolysis, accompanied by massive lipid droplet deposition, suggesting mitochondrial dysfunction and cardiomyocyte injury. Although multiple mechanisms can alter cardiac structure and function, our observation suggest that metabolic dysregulation may predominate in diabetic myocardial dysfunction. Notably, a study reported that systemic proprotein convertase subtilisin/kexin type 9 (PCSK9) deficiency reduced LDL-C levels but results in heart failure with preserved ejection fraction associated with changed in cardiac metabolism and increased cardiac accumulation of lipid droplets (LDs) [14]. These findings, in conjunction with ours, imply that LDL-C reduction strategies must consider potential myocardial metabolic disruptions to prevent adverse cardiac outcomes.

**SREBP1-activated lipogenesis is crucial for statins treatment-induced diabetes myocardial lipid deposition-**Our research revealed that SREBP activation was associated increase myocardial DNL and lipid deposition after long-term statins treatment in *db/db* mice, which was reversed by SREBP knockdown and inhibition. FFAs are the major substrate for ATP production in adult cardiac mitochondria, providing 60–70 % of the heart's energy [15]. Studies have indicated that in TIIDM, cardiomyocytes exhibit a reduced capacity to utilize fatty acids; this, coupled with increased FFA delivery, leads to intracellular lipid accumulation [16]. Such accumulation may precipitate mitochondrial dysfunction, ROS production, oxidative damage, and reduced ATP production, including sulfhydryl oxidation, lipid peroxidation, and mitochondrial DNA mutation [17]. Sustained DNL through the maintenance of active SRBEP1 is a key feature of lipid deposition in cells [12]. The principal physiological role of the SREBP1-mediated lipogenesis pathway is to facilitate DNL and energy storage. However, the capacity of cardiomyocytes to synthesize fatty acids remains a contentious issue. Early studies suggested that the cardiomyocyte had a marginal capacity for DNL, stimulated by succinate and mediated by the NADH: NAD^+^ ratio [18,19]. Under conditions such as fasting or diabetes, the heart could initiate FA synthesis in the mitochondrial system [20]. Failure of glycogen utilization during fasting and diabetes might be associated with the heart's unaltered lipogenetic activity. Our in *vitro* studies with NMPCs demonstrated that statin treatment promotes DNL under high-glucose conditions, further confirming increased FA synthesis in cardiomyocytes. Our research supported the hypothesis that in TIIDM state, cardiomyocyte possess minimal DNL activity, which can be augmented by statin therapy through SREBP1 activation, potentially resulting in myocardial lipid peroxidation and lipid deposition.

**SREBP1-related lipid peroxidation contributed to statins treatment-induced diabetes myocardial dysfunction-**In our study, we observed increased expression of the SREBP1 targeted lipogenesis pathway genes in the heart of statin-treated *db/db* mice. SREBP1-N was significantly elevated and translocated to the nucleus, with its target gene FASN, ACC1, and SCD1 synchronously upregulated. Elevated SREBP1 levels were correlated with myocardial lipid peroxidation, which was significantly alleviated by SREBP1 knockdown and inhibition in low dose STZ-induced TIIDM mice after statin therapy. SREBP1 activation has been associated with lipid accumulation and peroxidation in diabetic kidneys [21]. Lipid peroxidation can be described generally as a process under which oxidants such as free radicals attack lipids containing carbon-carbon double bond(s), especially polyunsaturated fatty acids (PUFAs) [22]. SREBP1-mediated lipid synthesis pathway upregulates PUFA synthesis [23], which make cells more susceptible to lipid peroxidation. Recent research elucidated how lipogenesis is nutritionally and differentially regulated by saturated and polyunsaturated fatty acids [24]. In general, saturated fatty acids can promote the activation of SREBP1, while unsaturated fatty acids inhibit the activation of SREBP1. SREBP1 predominantly orchestrates the synthesis of saturated fatty acids. Subsequently, these saturated fatty acids undergo transformation into their unsaturated counterparts under the catalytic influence of the enzyme SCD1 [25]. Our findings indicate that SREBP1 can mediate the synthesis of saturated fatty acids (Hexadecanoic acid and octadecanoic acid) in cardiomyocytes, suggesting that these saturated fatty acids may further activate SREBP1. As for the proportion of saturated fatty acids and unsaturated fatty acids in the myocardium and the effect on SREBP1, it is still unclear, which is the direction of our further research in the future. Myocardial lipid peroxidation is a significant etiological factor in cardiovascular complications associated with diabetes [26]. Prior research suggests that inhibition of the SREBP may mitigate myocardial lipid accumulation [27]. Our study delineates a novel mechanism by which SREBP inhibitory therapy could protect cardiac function in TIIDM by preventing lipid peroxidation.

**Benefit and mechanism of combined statin therapy-**under physiological conditions, the C-terminal domain of SREBP1 binds to SCAP in the ER membrane. When deprived of cholesterol and high cellular glucose [28], SCAP escorts SREBP1 to the Golgi for cleavage by S1P and S2P, releasing nSREBP1 for nuclear translocation and target gene activation. When cholesterol accumulates in the ER, the SCAP-SREBP complex is retained in the ER by binding to INSIG, blocking SREBP activation [29]. Three N-glycosylation sites on SCAP enhance its protein levels and promote SREBP1 activation [28]. Similar phenomena of increased lipogenesis and activated SREBP2 were observed in hyperphosphatemic chronic kidney disease mouse models [30]. We propose a hypothesis that statins may enhance SCAP N-glycosylation under high glucose, promoting SREBP1 activation. In diabetic cardiomyocytes, statin induced SCAP N-glycosylation, facilitating SCAP/SREBP trafficking and SREBP1 activation. Our further analysis in myocardial tissues from diabetic patients revealed high expression of SREBP1, correlated with myocardial fibrosis and lipid peroxidation. It is evident that our mechanistic investigations necessitate additional research. Specifically, we require further examination of the glycosylation sites on SCAP and the identification of the binding motif for INSIG or SCAP for N-linked glycosylation.

Previous research has demonstrated that l-carnitine treatment augments FAO and CPTI expression, furthermore l-carnitine supplementation might be an effective tool for improvement of glucose utilization in TIIDM [31], which is important in the pathogenesis of multiple cardiovascular disorders [32]. Notably, l-carnitine significantly down-regulated the expressions of SREBP1 [33]. We hypothesize that l-carnitine could serve as an efficacious intervention to counteract statin-induced intramyocardial lipid peroxidation and lipid accumulation, thereby enhancing FAO and glucose utilization. Our findings indicate that the concurrent use of statins and l-carnitine mitigated statin-induced myocardial dysfunction and pathological remodeling. Employing a combination therapy that targets both lipid and glucose metabolism might be an effective strategy to decrease the risk of cardiovascular events in patients undergoing lipid-lowering drug therapy.

In summary, our findings underscore that long-term statin therapy was associated with diabetic myocardial dysfunction. Clinicians should consider the metabolic effects and risk-benefit ratio when prescribing statin therapy in TIIDM patients. Our findings supported the use of statins in combination with other lipid-lowering or metabolic-improving drugs to reduce residual cardiovascular risk more effectively and safely. A limitation of this study is the absence of clinical outcomes related to the prolonged use of statins in T2DM patients. Future research should focus on elucidating the roles of glucose accumulation, SREBP1 activation, and SCAP N-glycosylation in the amelioration of statin-induced myocardial dysfunction. Additionally, research is needed to confirm cardiomyocyte fatty acid synthesis under specific pathological conditions in vivo. We contend that our findings enrich the current comprehension of the intricate interplay between statin therapy and myocardial lipid metabolism in the context of diabetes.

## 4 Methods

### 4.1 Human subjects

Heart samples from 10 healthy individuals and 12 TIIDM patients used in this study were collected from the National Center for Medico-legal Expertise of Sun Yat-sen University. The use of human heart samples was approved by the Ethics Committee of Zhongshan School of Medicine, Sun Yat-sen University (grant No.: 2019–004), and all data and sample collection were conducted in strict accordance with the ethics guidelines of Zhongshan School of Medicine, Sun Yat-sen University. Informed consent was obtained from the legal representatives of the victims. The principles outlined in the Declaration of Helsinki were followed. Please refer to Supplementary Table 2 for information about the decedents’ age, sex, and heart details.

### 4.2 Animal experiments

Four-week-old male C57BLKS/J^*db/db*^ (*db/db*) mice, age-matched male non-diabetic C57BLKS/J^*db/m*^ (*db/m*) mice were purchased from GemPharmatech Co., Ltd (Jiangsu, China) following breeding at the Center for Disease Model Animals of Sun Yat-sen University. Ten-weeks-old male *db/db* mice and *db/m* mice were enrolled in these experiments and divided into five groups: Db/m group, Db group, Db + Ato5 group (atorvastatin 5 mg kg^−1^ BW/day), Db + Ato10 group (atorvastatin10 mg kg^−1^ BW/day) and Db + Rosu20 group (rosuvastatin 20 mg kg^−1^ BW/day). The doses selected for our study were determined after review of the literature [[34], [35], [36]] and preliminary experiments conducted to establish the appropriate dosing range in mice that would yield therapeutic effects without causing undue toxicity. We initially dissolved rosuvastatin calcium in normal saline, prepared the appropriate concentration according to the dose. Then placed it in an ultrasonic drug dissolution instrument to fully shake, and at the same time heated at 37° to make rosuvastatin calcium dissolve as much as possible. Statins administered five consecutive days a week via oral gavage. In addition, we also used *db/m* mice as a control group administered statin and divided into four groups: Db/m group, Db/m + Ato5 group, Db/m + Ato10 group; Db/m + Rosu20 group. The drugs were dissolved in saline and administered through oral gavage once a day for 40 weeks. All animals were given water and chow diet during the whole experiment period. During this period, body weight, fasting blood glucose (FBG) were measured.

Low dose STZ-induced TIIDM mouse model was constructed using a method described previously in our previous publication [8,26]. Briefly, mice of the model group were treated with HFD at 4-weeks-old and were treated with seven consecutive intravenous injections of STZ (40 mg/kg, Sigma, St. Louis, MO) in citrate buffer (pH 4.6) at 8-weeks-old, while the control animals received chow diet and the same volume of citrate buffer. The animals of model group were given 10 % sucrose/water during the period from 12 h after the first STZ injection to 12 h after the last injection and were given HFD during the whole experiment period. The animals of control group were given water and chow diet during the whole experiment period. The blood glucose level was monitored with a glucometer (One Touch Ultra Easy, Life Scan, PA, USA), on 2 weeks after the last STZ injection, and animals with blood glucose levels greater than 12 mmol/L were considered diabetic.

Eight-week-old male C57BL/6 J and KK-Ay mice were obtained from Beijing HFK Bioscience Co. Ltd. (Beijing, China). KK-Ay mice were a kind of T2DM mouse model and allowed free access to HFD [37]. KK-Ay mice are a cross between diabetic KK and lethal yellow (Ay) mice, and carry a heterozygous mutation of the agouti gene. The severity of hyperglycemia and insulin resistance is exacerbated by the introduction of Ay allele into the KK background. The genetic background of KK mice and KK-Ay mice is the inbred mouse strain of C57BL/6 J, which is always used as the control for KK mice or KK-Ay mice. C57BL/6 J mice were fed regular chow and there were considered as control group. *n* = 4 in each group. Only male mice were enrolled. The experimental grouping, and statins treatment of KK-Ay mice and low dose STZ-induced TIIDM mouse model were the same as those of *db/db* mice. All animal experiments details were consistent with *db/db* mice.

SREBP1-KO mice (Strain NO. T037279, genetic background: C57BL/6 J) were purchased from GemPharmatech (Nanjing, China). Heterozygous F0 generation mice were obtained by freezing sperm resuscitation. After hybridization of F0 generation mice, F1 generation heterozygous mice (*SREBP1*^*+/−*^) were obtained for low dose STZ induce TIIDM model (8 weeks old). The experimental grouping, and statins treatment were the same as those of *db/db* mice.

Because of the possible effects of estrogens on the heart, only adult male mice were used for all experiments [26,38]. All mice were housed in an animal facility with a 12 h light–dark cycle and water. All animal experiments were performed according to the regulations approved by the Animal Care and Ethics Committee of Sun Yat-sen University (the protocol number is 2021000957).

### 4.3 Echocardiography

Mice were anesthetized with 1.5 % isoflurane and placed on a thermostat at 37 °C immediately, 0.5 % isoflurane is inhaled continuously to prevent them from waking up. The heart rates of the mice were controlled at 450–600 bpm. For image acquisition, Vevo 3100 Imaging System (VisualSonics, Canada) with a 400 MHz probe was used to detect cardiac motion in the long-axis view, then the probe was rotated 90° to detect cardiac motion in the short-axis view, and graphs were acquired in M-Mode near the papillary muscles.

### 4.4 Cardiomyocyte contractility assay

Following existing literature methods [39,40], we used the integrated IonOptix contractility/photometry system to measure sarcomere shortening and relaxation in freshly isolated left ventricular cardiomyocytes from mouse hearts. Cardiomyocytes were maintained in normal Tyrode solution after being restored to calcium ion concentration at room temperature. An electric field of 1 Hz was set with a myopacer (IonOptix MYP100) through platinum electrodes lowered into the bath for electrical stimulation of cardiomyocytes while tracking sarcomere transient traces and sarcomere length changes. Basal and peak sarcomere lengths, maximum departure and return velocities, and time to peak or to baseline were measured. All measurements were performed at room temperature within 3 h. Data were collected and analyzed using IonWizard 7.4.

### 4.5 Assessment of indirect calorimetry

Indirect calorimetry data were recorded in the studied mice by using a Promethion Metabolic Cage System (Sable Systems, USA) as described previously [41]. Mice were acclimated for 2 days in metabolic cages before recording calorimetric variables. Animals were housed individually in metabolic chambers maintained at 28 °C under a 12 h light/dark cycle with free access to food and water. Oxygen consumption (VO_2_) and carbon dioxide production (VCO_2_) for individual mice were measured. The respiratory exchange ratio (RER) was calculated as VCO_2_/VO_2_. Other indicators calculated as described previously [41].

### 4.6 Transmission electron microscopy (TEM)

Cardiac ultrastructure was examined under a transmission electron microscope (Tecnai G2 SpiritTwin + GATAN 832.10 W, FEI, Czech) using conventional methods. In brief, heart tissues were fixed with 2.5 % glutaraldehyde in 0.1 mol/L phosphate buffer (pH 7.4), followed by 1 % OsO4. After dehydration, thin sections were stained with uranyl acetate and lead citrate for observation, images were acquired digitally.

### 4.7 Histological analysis and oil red O staining

The hearts were harvested and fixed overnight in 4 % paraformaldehyde and dehydrated with alcohol for paraffin embedding. Heart sections (4 μm) were stained with H&E, Masson's trichrome, Sirius red, Periodic Acid-Schiff (PAS) stain according to standard protocols. All the histological staining images were captured by a microscope (DFC700T, Leica, Germany).

For Oil Red O staining, the heart tissues were fixed in 4 % paraformaldehyde (Biosharp, Hefei, China) overnight then dehydrated with a sucrose gradient, and embedded in the Tissue-Tek OCT compound (Sakura Finetek, Tokyo, Japan). Then, sections (7 μm) were stained with oil red O (G1261, Beijing Solarbio Science & Technology Co., Ltd., Beijing, China) for 20 min.

### 4.8 Immunohistochemistry (IHC) and immunofluorescence (IF)

The heart samples sections were blocked with 3 % hydrogen peroxide and then performed at 95 °C for 10min using citrate buffer (Beyotime, Shanghai, China), then blocking steps were carried out using the QuickBlock™ Blocking Buffer (Beyotime, Shanghai, China) according to the manufacturer's instructions. The sections were then incubated with antibodies. After incubated with primary antibody at 4 °C overnight, the sections incubated with secondary antibody at 37 °C for 30min. Visualization was accomplished using 3,3N-diaminobenzidine tertrahydrochloride (DAB). In the negative controls, the primary antibody was omitted and replaced with the blocking solution. All the histological staining and immune-fluorescence images were captured by a microscope (DFC700T, Leica, Germany). The positive area was quantified with the ImageJ software program (National Institutes of Health, Bethesda, MD, USA). The antibodies are listed in Supplementary Table 3.

### 4.9 Immunoblot analysis

For western blotting analysis, an equal number of proteins (30 mg) was resolved by an 8–12 % gel, transferred to polyvinylidene fluoride (PVDF) membranes (Millipore, Burlington, MA, USA), and incubated with appropriate primary antibodies at 4 °C overnight. Membranes were washed three times by Tris-buffered saline containing 0.1 % tween-80 (TBST) and incubated with peroxidase-conjugated secondary antibodies. Protein bands were visualized with the enhanced chemiluminescence (ECL) reagent using a ChemiDoc MP Imaging System (Bio-Rad Co., Hercules, CA, USA). The antibodies are listed in Supplementary Table 3.

### 4.10 Serum/tissue measurement

The serum brain natriuretic peptide (BNP) levels were examined using an enzyme-linked immunosorbent assay (ELISA) (CSB-E07971 m, Cusabio, Wuhan, China). The heart tissue TG, TCHO, LDL and HDL levels were examined using commercial reagent kits (Jiancheng, Nanjing, Jiangsu, China). The heart and liver tissue glucose levels were examined using commercial reagent kits (ADS078TC0, Jiangsu Meimian Industrial Co., Ltd, Jiangsu, China).

### 4.11 Vitro experiment groups for Western blot analysis and IF

NMPCs were subjected to starvation in a serum-free medium overnight, then divide into 6 groups, respectively cultured in glucose 1.0 mmol/L (group 1), cultured in a glucose 4.5 mmol/L (group 2), the group 3 treated with glucose 4.5 mmoL/l and atorvastatin 10 μmol/L (Sigma-Aldrich, PHR1422), the group 4 treated with tunicamycin (1 μg/mL) (ab120296, Abcam), the group 5 treated with GlcNAc (20 mM) (S6257, Selleck Chemicals), the group 6 treated with glucose 4.5 mmol/L and atorvastatin10 mmol/l, tunicamycin (1 μg/mL). All groups NMPCs were cultured for 24 h. The experiment was repeated three times.

### 4.12 Detection of SCAP N-glycosylation

The human AC-16 cardiomyocytes were subjected to starvation in a serum-free medium overnight, then divide into 6 groups, respectively cultured in glucose 1.0 mmol/L (group 1), cultured in a glucose 4.5 mmol/L (group 2), the group 3 treated with glucose 4.5 mmol/L and atorvastatin 10 μmol/L, the group 4 and 5 treated with tunicamycin (1 μg/mL), the group 6 and 7 treated with glucose 4.5 mmoL/l and atorvastatin 10 μmol/L and tunicamycin. All groups AC-16 cardiomyocytes were cultured for 24 h. And then detection of SCAP N-glycosylation. The groups of 1, 2, 3, 5, and 7 subsequent treatments with PNGase F. The experiment was repeated three times.

The detection of SCAP N-glycosylation was performed according to the method described previously [28,42]. Briefly, the membrane pellets from cell lysates prepared as described above were resuspended in 114 μL of buffer containing 10 mM HEPES·KOH (pH 7.4), 10 mM KCl, 1.5 mM MgCl_2_, 1 mM sodium EDTA, and 100 mM NaCl. Aliquots of membrane proteins were then incubated in the absence or presence of 1 μg of trypsin (T6567, Sigma), in a total volume of 58 μL, for 30 min at 30 °C. Reactions were stopped by the addition of 2 μL (400 units) of soybean trypsin inhibitor (T9777, Sigma). The samples were then heated at 100 °C for 10 min with 5x loading buffer and subjected to SDS-PAGE. Combine 1–20 μg of glycoprotein, 1 μL of Glycoprotein Denaturing Buffer (10X) and H_2_O (if necessary) to make a 10 μL total reaction volume. Denature glycoprotein by heating reaction at 100 °C for 10 min. Chill denatured glycoprotein on ice and centrifuge 10 s. Make a total reaction volume of 20 (40) μL by adding 2 μL GlycoBuffer 2 (10x), 2 μL 10 % NP-40 and 6 μL H2O. Add 1 μL PNGase F, mix gently. Incubate reaction at 37 °C for 1 h. The mixtures were heated at 100 °C for 10 min and subjected to SDS-PAGE.

### 4.13 Statistical analysis

All statistical analysis was performed using Prism 9 (GraphPad Software Inc) and IBM SPSS Statistics software (SPSS) (Versions 22.0) (Inc., Chicago, IL). Data are expressed as mean ± SEM. All experiments were repeated at least 3 times with representative data shown. A two-tailed unpaired Student t-test was performed to determine the difference between 2 groups. For comparisons of ≥3 groups, two-tailed, One-way ANOVA followed by Tukey's test was performed. For correlation analysis, linear regression models were performed and the goodness of fit for regression models was assessed using R values. All group numbers and detailed significant values were presented within the figure or their legends. *P* values < 0.05 were considered to indicate statistically significant differences.

## CRediT authorship contribution statement

**Tong-sheng Huang:** Conceptualization, Data curation, Formal analysis, Funding acquisition, Investigation, Methodology, Project administration, Resources, Writing – original draft, Writing – review & editing. **Teng Wu:** Data curation, Formal analysis, Methodology, Project administration. **Xin-lu Fu:** Investigation, Methodology, Project administration. **Hong-lin Ren:** Investigation, Methodology, Resources. **Xiao-dan He:** Formal analysis, Methodology. **Ding-hao Zheng:** Data curation, Formal analysis, Methodology. **Jing Tan:** Formal analysis, Funding acquisition, Investigation. **Cong-hui Shen:** Data curation, Formal analysis. **Shi-jie Xiong:** Data curation, Formal analysis. **Jiang Qian:** Data curation. **Yan Zou:** Data curation. **Jun-hong Wan:** Formal analysis, Investigation. **Yuan-jun Ji:** Methodology. **Meng-ying Liu:** Data curation. **Yan-di Wu:** Data curation. **Xing-hui Li:** Data curation. **Hui Li:** Data curation, Formal analysis, Investigation. **Kai Zheng:** Writing – review & editing. **Xiao-feng Yang:** Supervision, Writing – review & editing. **Hong Wang:** Supervision, Writing – review & editing. **Meng Ren:** Funding acquisition, Supervision. **Wei-bin Cai:** Data curation, Funding acquisition, Project administration, Supervision, Writing – review & editing.

## Data availability statement

Source data contained the raw data underlying the following types of display items: any reported means/averages in bar charts, and tables, and uncropped versions of any gels or blots, labeled with the relevant panel and identifying information. Source data are provided with this paper. The data that support the findings of this study are available from the corresponding author upon reasonable request.

## Declaration of competing interest

The authors declare that they have no known competing financial interests or personal relationships that could have appeared to influence the work reported in this paper.
