# Supplementary material for: SREBP1 induction mediates long-term statins therapy related myocardial lipid peroxidation and lipid deposition in TIIDM mice
Source: Redox Biol. 2024 Oct 28;78:103412. doi: 10.1016/j.redox.2024.103412 (PMC11555471; doi:10.1016/j.redox.2024.103412)
Supplement: Multimedia component 1 [file mmc1.docx]

**Supplementary Materials for**

**SREBP1 induction mediates** **long-term statins therapy related myocardial lipid peroxidation and lipid deposition in TIIDM mice**

Tong-sheng Huang ^1,2^ **^#^**, Teng Wu^1,2^ **^#^**, Xin-lu Fu^1,2^, Hong-lin Ren^1,2^, Xiao-dan He^3,4^, Ding-hao Zheng^3,4^, Jing Tan ^1,2^, Cong-hui Shen^1,2^, Shi-jie Xiong^1,2^, Jiang Qian^1,2^, Yan Zou^1,2^, Jun-hong Wan^1,2^, Yuan-jun Ji^1,2^, Meng-ying Liu ^1,2^, Yan-di Wu^1,2^, Xing-hui Li ^1,2^, Hui Li^1^, Kai Zheng^5^, Xiaofeng Yang^4^, Hong Wang^4^*****, Meng Ren^3^*****, Wei-bin Cai^1,2^ *****

^1^Guangdong Engineering & Technology Research Center for Disease-Model Animals, Laboratory Animal Center, and ^2^Department of Biochemistry, Zhongshan School of Medicine, Sun Yat-sen University, Guangzhou 510080, Guangdong, China.

^3^Department of Endocrinology, Sun Yat-sen Memorial Hospital, Sun Yat-sen University, Guangzhou, China.

^4^Metabolic Disease Research, Department of Cardiovascular Sciences, Temple University Lewis Katz School of Medicine, Philadelphia, PA, USA.

^5^School of Biomedical Engineering, Shenzhen Campus of Sun Yat-sen University, Shenzhen 518107, Guangdong, China.

^#^ These authors contributed equally: Tong-sheng Huang, Teng Wu.

***Correspondence to:**

Wei-bin Cai, Sun Yat-sen University, Guangzhou 510080, PR China (Email: caiwb@mail.sysu.edu.cn).

Meng Ren, Department of Endocrinology, Sun Yat-sen Memorial Hospital, Sun Yat-sen University, Guangzhou, China (Email: renmeng80@139.com).

Hong Wang, Temple University Lewis Katz School of Medicine, Philadelphia, PA, USA (Email: [hong.wang@temple.edu](mailto:hong.wang@temple.edu)).

This PDF file includes:

Supplementary Table 1 to 3;

Supplementary Figure 1 to 9.

**Supplementary Tables**

**Supplementary Table 1.** **Clinical information for collected human heart samples.** Abbreviations: LV, left ventricle. RV, right ventricle. Data were expressed as means ± SEM.

| Category | Normal (n=10) | T2DM (n=12) | *P* value |
| --- | --- | --- | --- |
| Age, years | 46.1±6.14 | 42.67±4.86 | 0.662 |
| Gender, Male % | 60% | 66.7% | - |
| Heart weight, g | 298.00±18.90 | 362.50±26.80 | 0.073 |
| LV thickness, cm | 1.12±0.04 | 1.21±0.34 | 0.121 |
| RV thickness, cm | 0.27±0.02 | 0.28±0.02 | 0.568 |

| Co-morbidities | | | |
| --- | --- | --- | --- |
|  | Normal (n=10) |  | T2DM (n=12) |
| Normal-1 | None | Patient-1 | TIIDM |
| Normal-2 | Omarthritis | Patient-2 | TIIDM, pancreatitis |
| Normal-3 | Necrotic drug eruption | Patient-3 | TIIDM, supraventricular arrhyth- mia |
| Normal-4 | None | Patient-4 | TIIDM |
| Normal-5 | None | Patient-5 | TIIDM, arrhythmia |
| Normal-6 | Chronic bronchitis | Patient-6 | TIIDM, mental disorders |
| Normal-7 | None | Patient-7 | TIIDM, ovarian cyst |
| Normal-8 | None | Patient-8 | TIIDM, fatty liver |
| Normal-9 | None | Patient-9 | TIIDM, pyelonephritis |
| Normal-10 | None | Patient-10 | TIIDM |
|  |  | Patient-11 | TIIDM, hypertriglyceridemia |
|  |  | Patient-12 | TIIDM |

**Supplementary Table 2. Echocardiographic parameters of *db/db* mice in different groups.** **(See Fig. 3B)** Abbreviations: LV, Left Ventricular; LVAW, left ventricular anterior wall; LVPW, LV posterior wall thickness. *n* = 6 in each group. Results are presented as the means ± SEM.

| Groups | Db/m | Db/db | Db+Ato5 | Db+Ato10 | Db+Rosu20 |
| --- | --- | --- | --- | --- | --- |
| N | 6 | 6 | 6 | 6 | 6 |
| Diameter;s (mm) | 1.65±0.27 | 1.91±0.14 | 2.21±0.18^*^ | 2.18±0.13 | 2.03±0.19 |
| Diameter;d (mm) | 3.13±0.22 | 3.37±0.23 | 3.53±0.09 | 3.23±0.11 | 3.28±0.17 |
| Volume;s (μL) | 9.91±4.02 | 12.15±2.00 | 17.40±3.64* | 16.40±2.66* | 14.33±2.54 |
| Volume;d (μL) | 40.45±7.14 | 48.27±6.70* | 52.13±3.25 | 42.51±3.60 | 44.53±5.54 |
| Stroke Volume (μL) | 30.54±4.05 | 36.11±4.79* | 34.73±1.81 | 26.11±2.94 | 30.20±3.39 |
| Ejection Fraction (%) | 78.64±5.33 | 75.28±1.07 | 67.77±14.89 | 61.42±5.06^**#^ | 69.09±4.15 |
| Fractional Shortening (%) | 48.24±5.53 | 43.22±0.81 | 37.65±3.68 | 32.66±3.40^**#^ | 38.63±3.88 |
| Cardiac Output (mL/min) | 15.14±1.19 | 14.66±2.11 | 15.77±1.53 | 9.74±1.11^*#^ | 13.73±1.56 |
| LV Mass (mg) | 142.63±10.56 | 129.96±10.94 | 110.99±11.77^*^ | 103.18±8.34^*#^ | 106.10±10.25^*^ |
| LV Mass Cor (mg) | 114.10±8.45 | 103.97±8.75 | 88.79±9.41^*^ | 82.54±6.67^*^ | 84.88±8.20^*^ |
| LVAW; s (mm) | 1.80±0.10 | 1.56±0.10 | 1.37±0.17^**^ | 1.22±0.04^***#^ | 1.43±0.08^*^ |
| LVAW;d (mm) | 1.24±0.05 | 1.03±0.09* | 0.83±0.09^***^ | 0.93±0.05 | 0.94±0.10 |
| LVPW; s (mm) | 1.63±0.16 | 1.44±0.13 | 1.24±0.22 | 1.21±0.18 | 1.25±0.11 |
| LVPW;d (mm) | 1.12±0.08 | 1.05±0.17 | 0.94±0.17 | 0.92±0.12 | 0.91±0.08 |

****P*<0.001, ***P*<0.01, **P*<0.05 vs. respective Db/m group; *^#^P*<0.05 vs. respective Db group.

**Supplementary Table 3.** **Antibody Information.**

| **Antibodies** | **Manufacture and item NO.** | **Application** |
| --- | --- | --- |
| COL1A1 | Cell signaling technology (72026S) | IHC |
| CD68 | Cell signaling technology (97778S) | IHC |
| 4-HNE | Abcam (ab46545) | IHC |
| IL-1β | Cell signaling technology(12242S) | IHC |
| SREBP2 | Novus Biologicals (NB100-74543) | WB |
| SREBP1 | Novus Biologicals (NB100-2215) | WB, IHC，IF |
| FAS | Cell signaling technology (3180S) | WB, IHC |
| SCD1 | Cell signaling technology (2794S) | WB, IHC |
| ACC1 | Proteintech (21923-1-AP) | WB |
| ACC2 | Santa cruz biotechnology (sc-390344) | WB |
| ATGL (30A4) | Cell signaling technology (2439S) | WB |
| CD36 | Sigma aldrich (HPA002018) | WB, IHC |
| CPT1B | Proteintech (22170-1-AP) | WB |
| RAGE | Proteintech (16346-1-AP) | IHC |
| Phospho-Akt (Ser473) | Cell signaling technology (4060S) | WB |
| Phospho-p70 S6 Kinase (Thr389) | Cell signaling technology (9205S) | WB |
| p70(S6K) | Proteintech (14485-1-AP) | WB |
| GYS1 | Proteintech (10566-1-AP) | WB |
| Hexokinase 2 | Proteintech (66974-1-Ig) | WB |
| PKM2 (D78A4) | Cell signaling technology (4053S) | WB |
| PFKM | Proteintech (55028-1-AP) | WB |
| SCAP Antibody | Affinity Biosciences (DF13713) | IF |
| SCAP Antibody | Abcam (ab153933) | WB |
| SCAP (9D5) | Santa cruz biotechnology (sc-13553) | WB |
| INSIG-1 | Santa cruz biotechnology (sc-390504) | WB |
| GOLGIN-97 | Invitrogen(A21270) | IF |
| PDI | Proteintech (66422-1-Ig) | IF |
| Troponin T | Thermo Fisher Scientific (MA5-12960) | IF |
| HSP90 | Proteintech (13171-1-AP) | WB |

**Supplementary Figure and Figure legends**


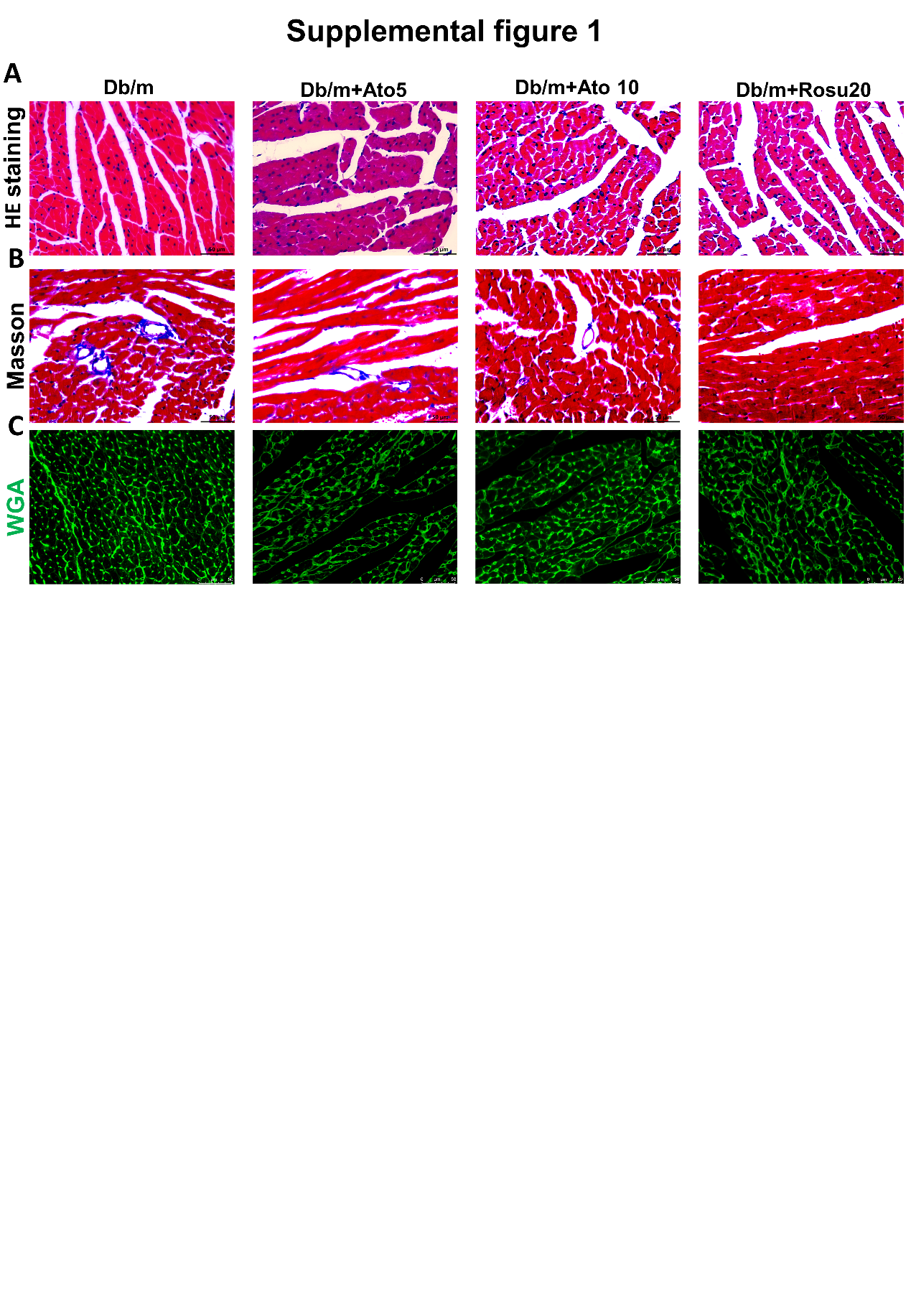


**Supplementary Figure 1.** **Pathological characteristics of long-term statins administration in the heart of *db/m* mice.**

**(A)** HE staining of heart from each group of *db/m* mice. **(B)** Representative Masson’s trichrome staining. **(C)** Representative FITC-conjugated WGA staining. Original magnification × 400, scale bar = 50 μm.


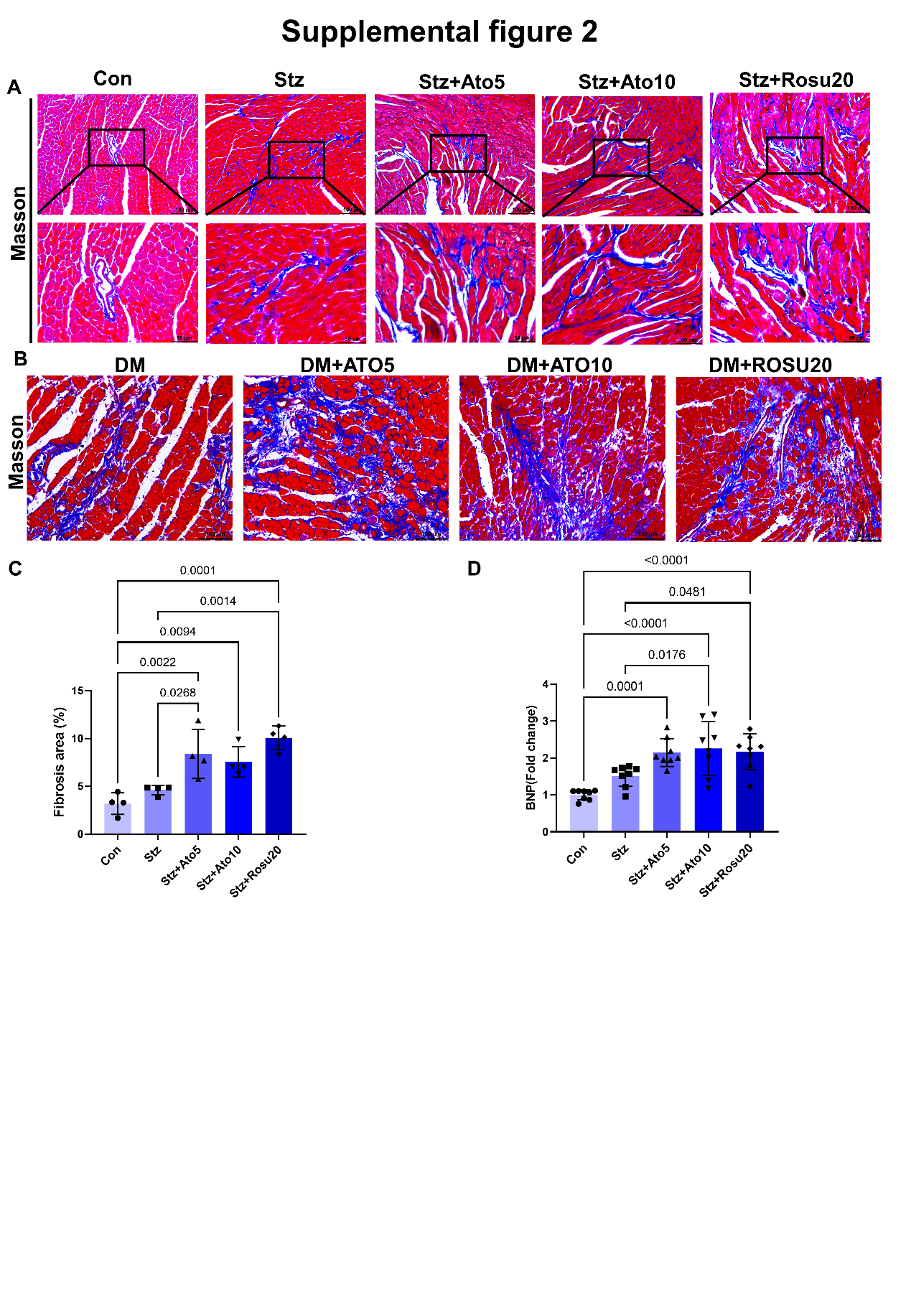


**Supplemental Figure 2. Long-term statins administration worsens cardiac dysfunction in low doses STZ-induced TIIDM mice and KK-ay TIIDM mice.**

**(A)** Masson’s trichrome staining of heart in statin treated low doses STZ-induced TIIDM mice. Original magnification × 200 or 400, scale bar = 100 or 50 μm. **(B)** Masson’s trichrome staining of heart in statin treated KK-ay TIIDM mice. Original magnification 400, scale bar = 50 μm. **(C)** Quantification of fibrosis area in the heart in statin treated low doses STZ-induced TIIDM mice. *n* = 4 in each group. **(D)** Detection of BNP in serum in statin treated in statin treated low doses STZ-induced TIIDM mice. *n* = 8 in each group. Data are expressed as means ± SEM. One-way ANOVA with Tukey post hoc test was used for the analysis of statistical significance.


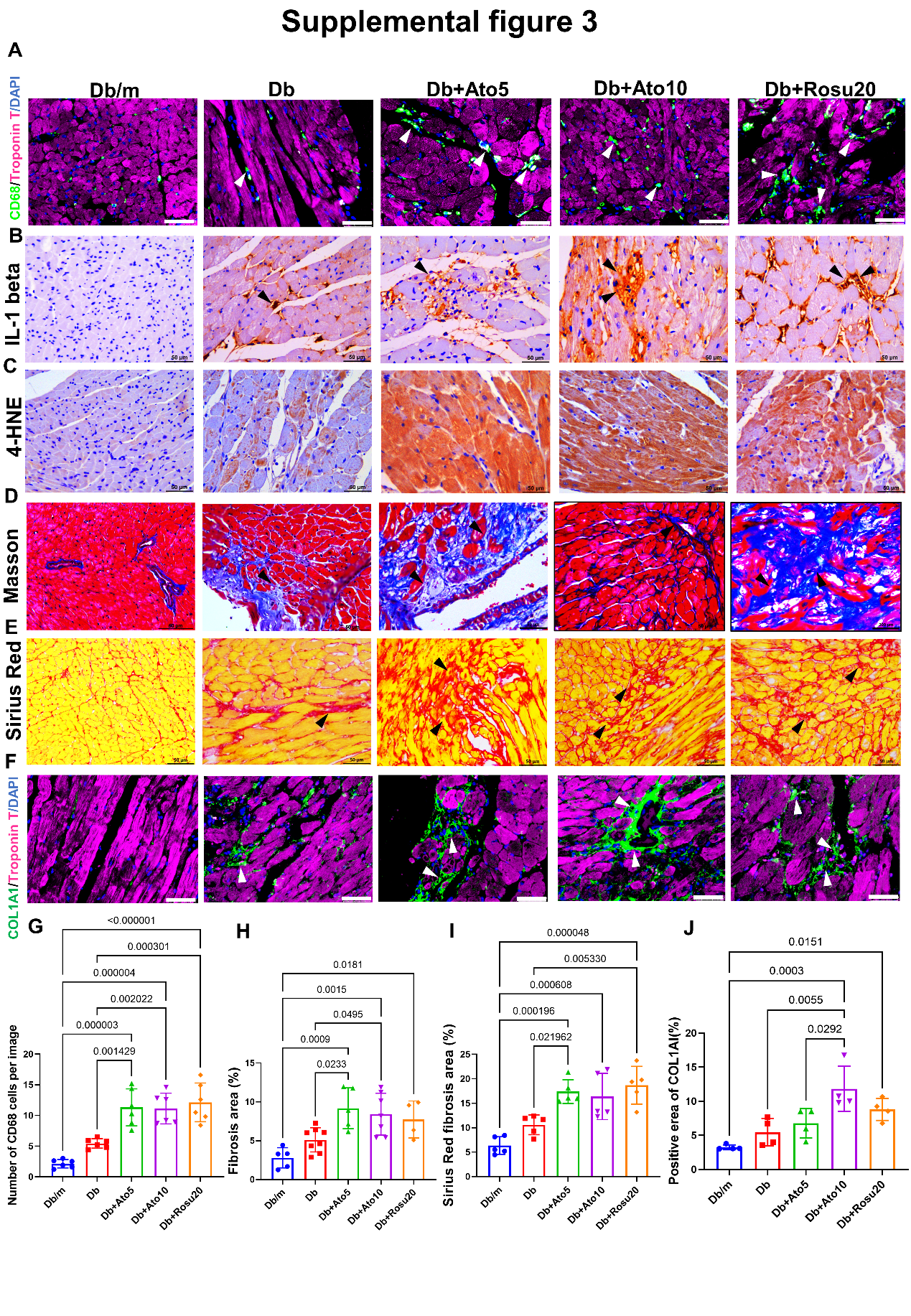


**Supplemental Figure 3.** **Long-term statins administration accelerates cardiac fibrosis and inflammation in *db/db* mice.**

**(A)** Immunofluorescence staining of macrophages marker protein CD68 (green) and cardiomyocyte marker Troponin T(pink) in the myocardium. White arrows indicated CD68-positive cells. Original magnification 1000, scale bar = 25 μm. **(B)** Immunohistochemistry staining of IL-1beta in the myocardium from each group of *db/db* mice. Black arrows indicated IL-1beta positive expression in the myocardium. Original magnification 400, scale bar = 50 μm. **(C)** Immunohistochemistry staining of 4-HNE in the myocardium from each group of *db/db* mice. Original magnification 400, scale bar = 50 μm. **(D-E)** Representative Masson’s trichrome staining and Sirius Red staining images in the myocardium. Black arrows indicated collagen deposition of heart sections. Original magnification 400, scale bar = 50 μm. **(E)** Immunofluorescence staining of protein COL1A1 (green) and cardiomyocyte marker Troponin T(pink) in the myocardium. White arrows indicated Type I Collagen deposition of heart sections. Original magnification 1000, scale bar = 25 μm. **(F)** The number of CD68-positive macrophage.  **(G-I)** Semiquantification analysis of fibrotic areas in heart sections. Data are expressed as means ± SEM. *n* = 6 in each group. One-way ANOVA with Tukey post hoc test was used for the analysis of statistical significance.


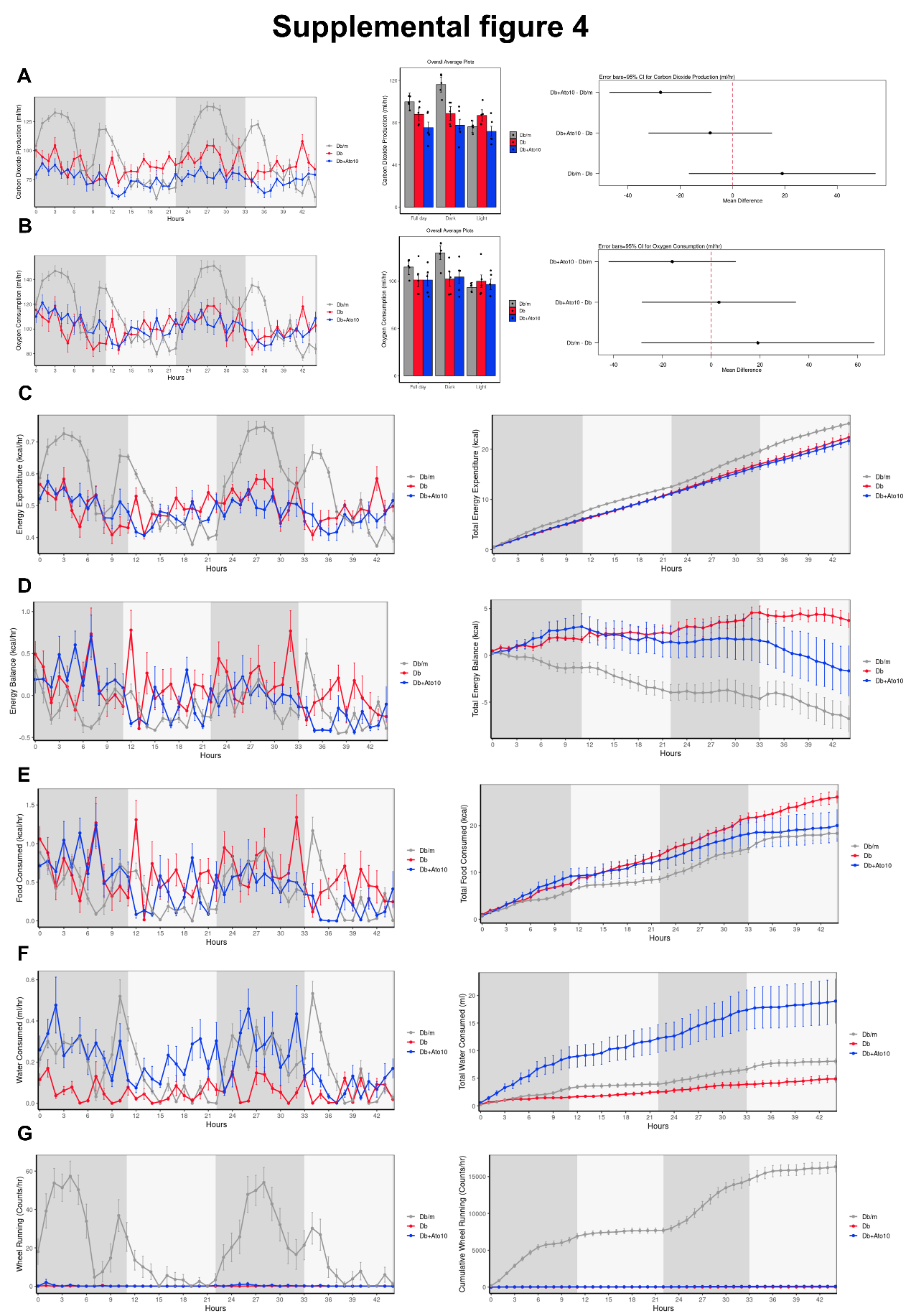


**Supplemental Figure 4. Metabolic measurements feature in statins therapy *db/db* mice.**

**(A)** Carbon dioxide production. **(B)** Oxygen consumption. **(C)** Energy expenditure. **(D)** Energy balance. **(E)** Food consumed. **(F)** Water consumed. **(G)** Wheel running. Data are expressed as means ± SEM. *n* = 4 in each group. One-way ANOVA with Tukey post hoc test was used for the analysis of statistical significance.


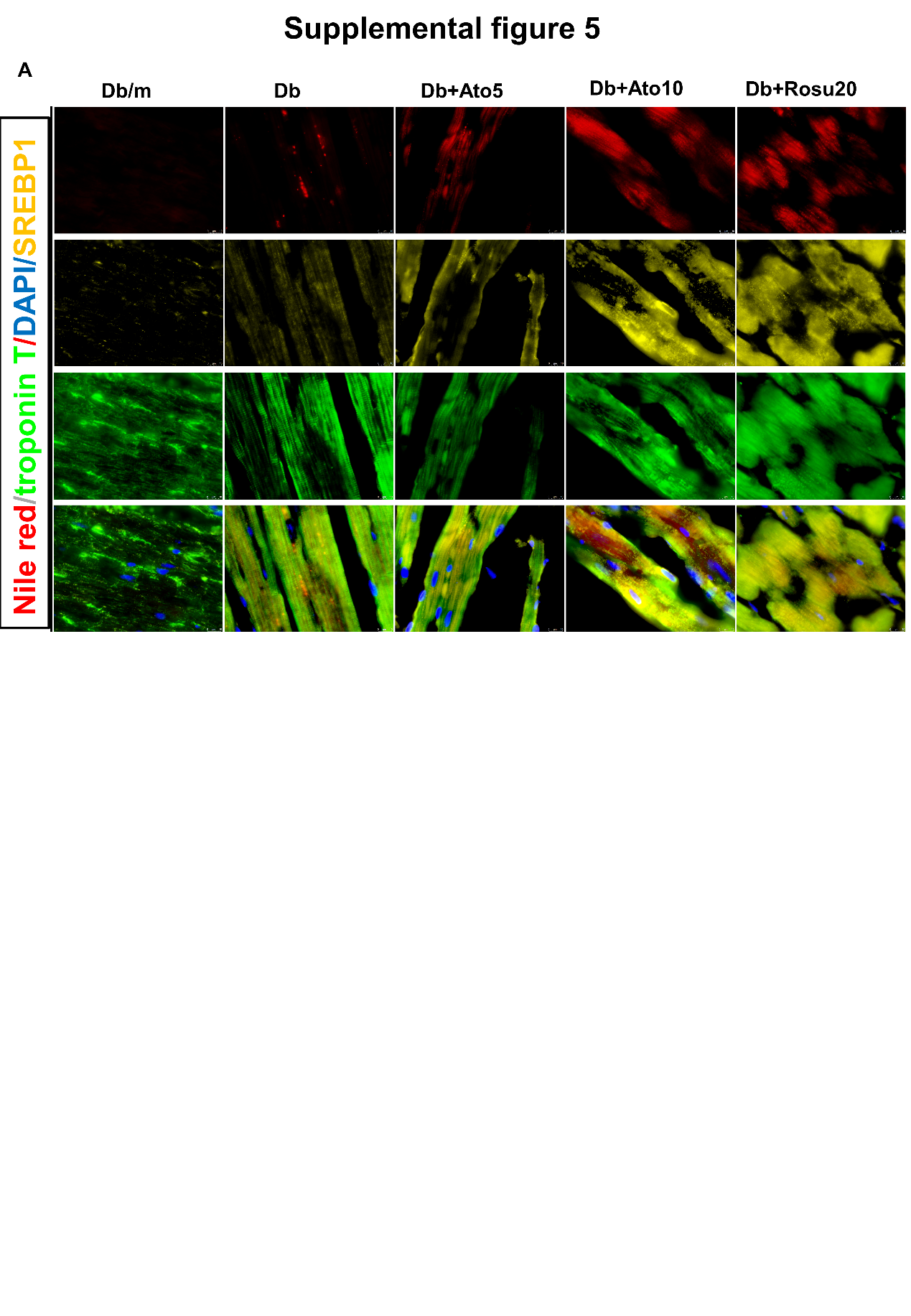


**Supplemental figure 5. The stimulation of SREBP-1 expression by statins accompanied by lipid deposition in the myocardium.**

**(A)** Immunofluorescence staining of protein SREBP1 (yellow), Nile red (red) and cardiomyocyte marker Troponin T (green) in the myocardium. White arrows indicated lipids in the myocardium, red arrows indicated SREBP1 nuclear translocation accompanied by lipid deposition in the myocardium. Original magnification 1000, scale bar = 25 μm.


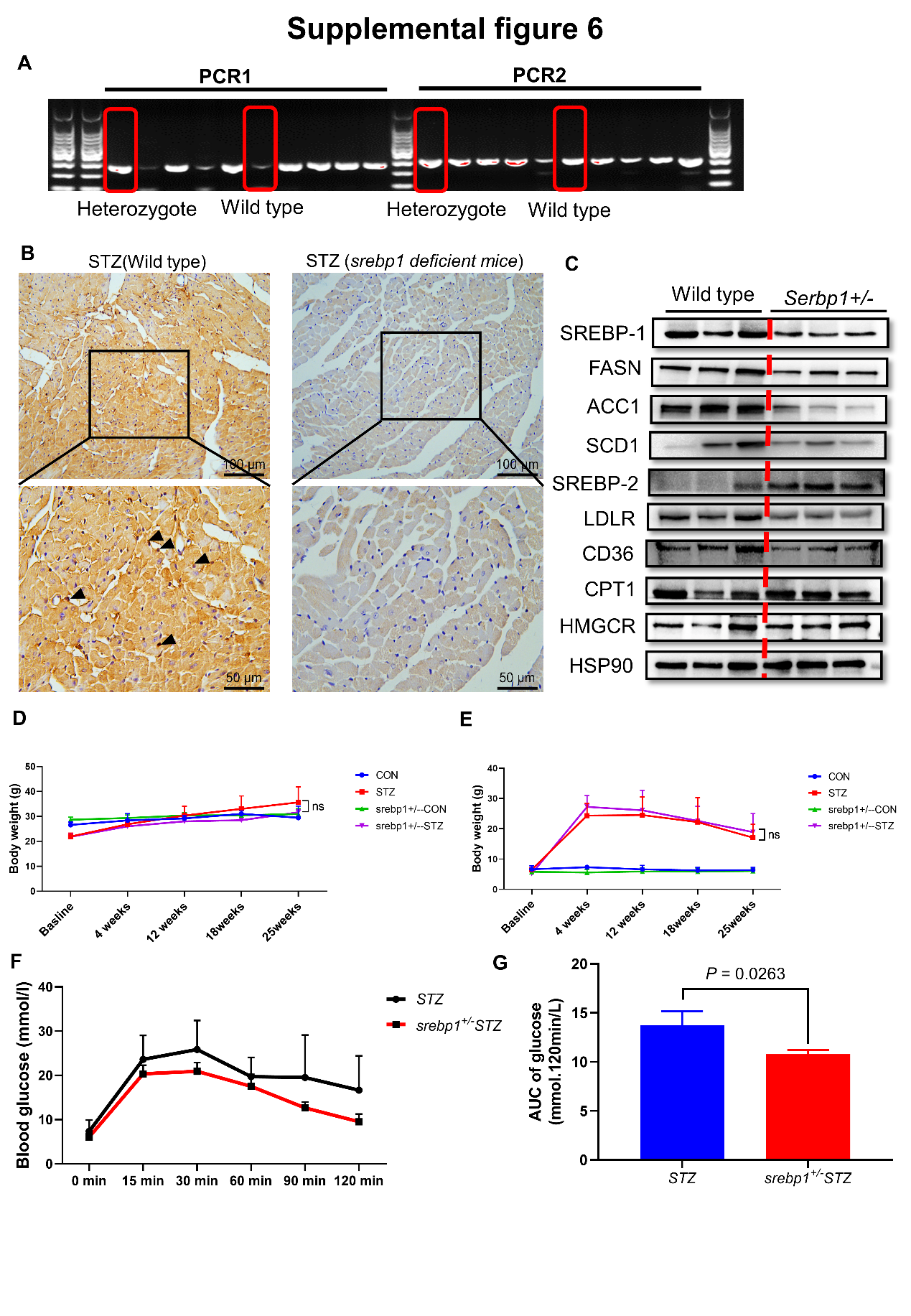


**Supplemental Figure 6. Genotyping and features of the *srebp1* deficient mouse and low doses STZ-induced srebp1-deficient TIIDM mice.**

**(A)** PCR analysis with mice tail tip DNA showing genotypes of srebp1 heterozygous and wild-type mice. The wild-type allele is represented by a band at 300 bp in PCR2 gel, whereas the disrupted allele shows 1 band at 300 bp in the PCR1 gel and 1 band at 300 bp in the PCR2 gel. **(B)** Immunohistochemistry staining of heart shown the expression of srebp1 was decreased in low doses STZ-induced srebp1 deficient TIIDM mouse. Original magnification × 200 or × 400, scale bar = 100 or 50 μm. **(C)** Western blot analysis srebp1 and related lipid metabolism gene of heart in wild-type and srebp1 deficient mice. **(D-E)** Body weight and fasting blood glucose levels for low dose STZ-induced SREBP1-deficient TIIDM mice (30 weeks). **(F-G)** Glucose tolerance test (GTT) and AUC for low dose STZ-induced SREBP1-deficient TIIDM mice (30 weeks). *n* = 4-6 in each group. Data are expressed as means ± SEM. One-way ANOVA with Tukey post hoc test or t-test were used for the analysis of statistical significance.


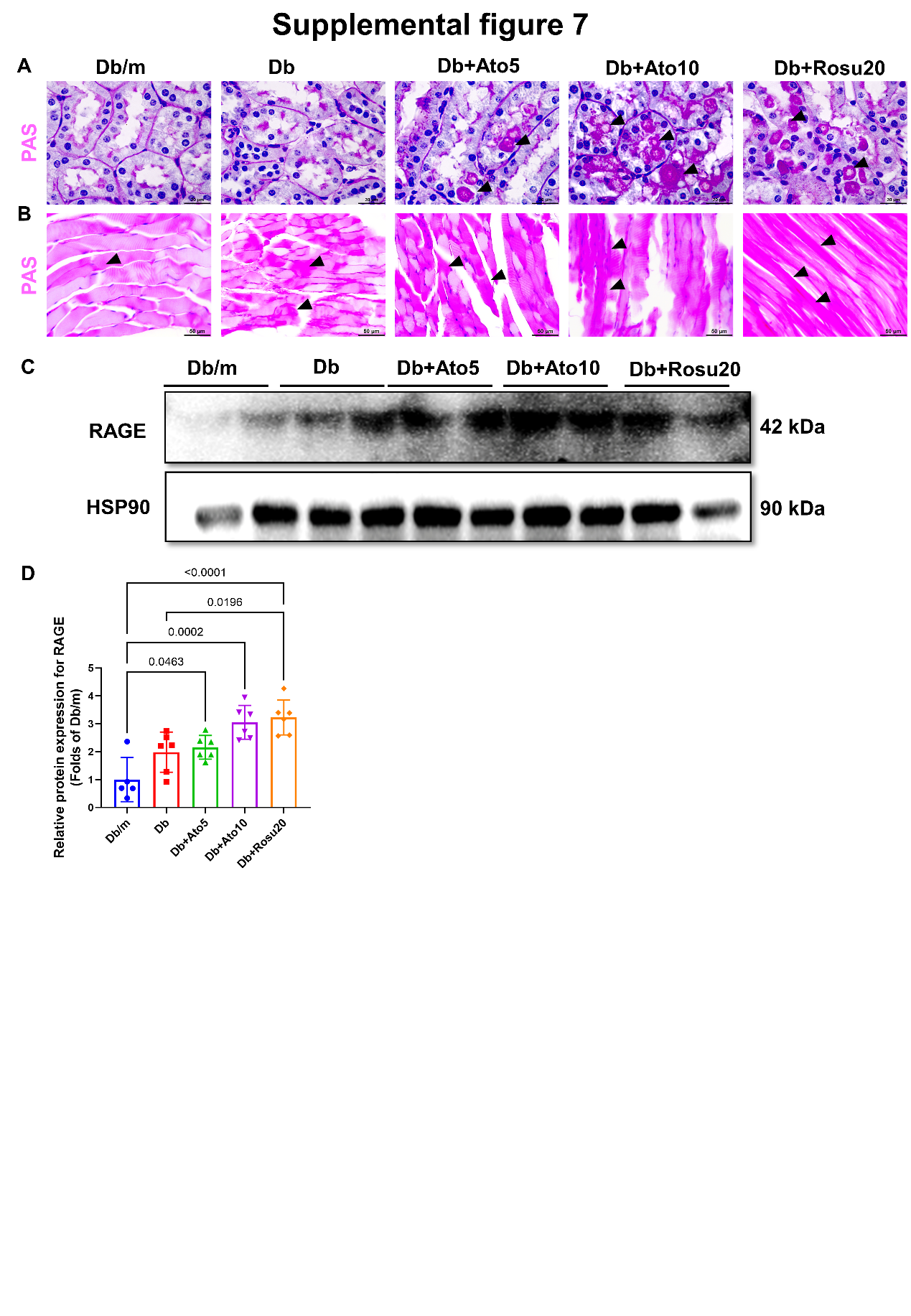


**Supplemental Figure 7. Long-term statins administration leads to muscle and kidney glycogen deposition.**

**(A)** PAS staining of kidney from each group of *db/db* mice. Black arrows indicated glycogen deposition in renal tubular epithelial cells. Original magnification × 1000, scale bar 20 μm. **(B)** PAS staining of muscle from each group of *db/db* mice. Black arrows indicated glycogen deposition in myocyte. Original magnification × 400, scale bar 50 μm. **(C-D)** Representative immunoblot images and quantification of RAGE in the heart tissues. HSP90 was used as an internal control. *n* = 6 in each group. Data are expressed as means ± SEM. One-way ANOVA with Tukey post hoc test was used for the analysis of statistical significance.


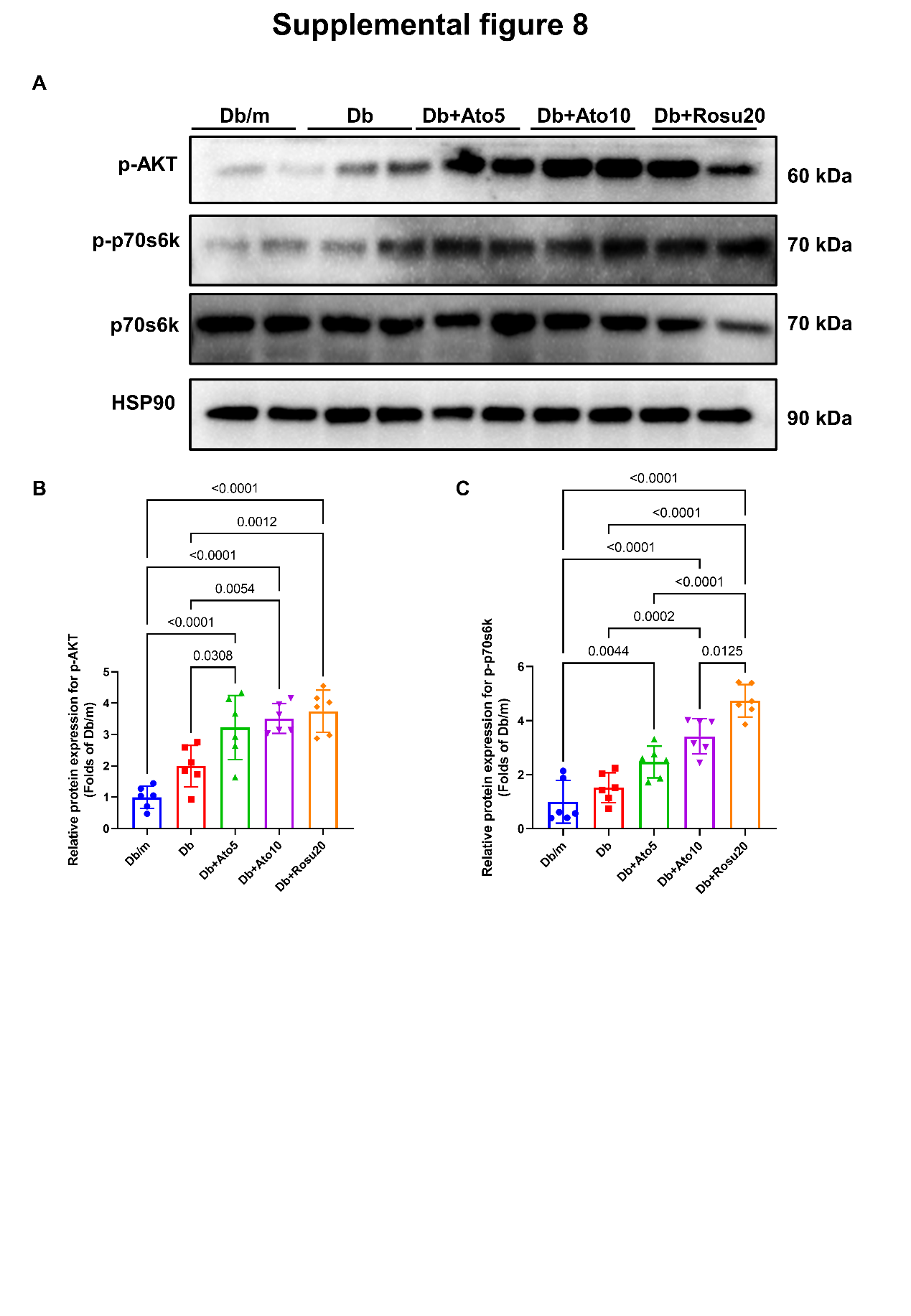


**Supplemental Figure 8. Long-term statins administration** **up-regulation of SREBP via AKT/mTOR Pathway.**

**(A)** Representative immunoblot images of p-AKT, p-p70s6k, and p70s6k in the heart tissues. HSP90 was used as an internal control. **(B-C)** Quantification of p-AKT, p-p70s6k, and p70s6k protein expression in the myocardium according to immunoblot. *n* = 6 in each group. Data are expressed as means ± SEM. One-way ANOVA with Tukey post hoc test was used for the analysis of statistical significance.


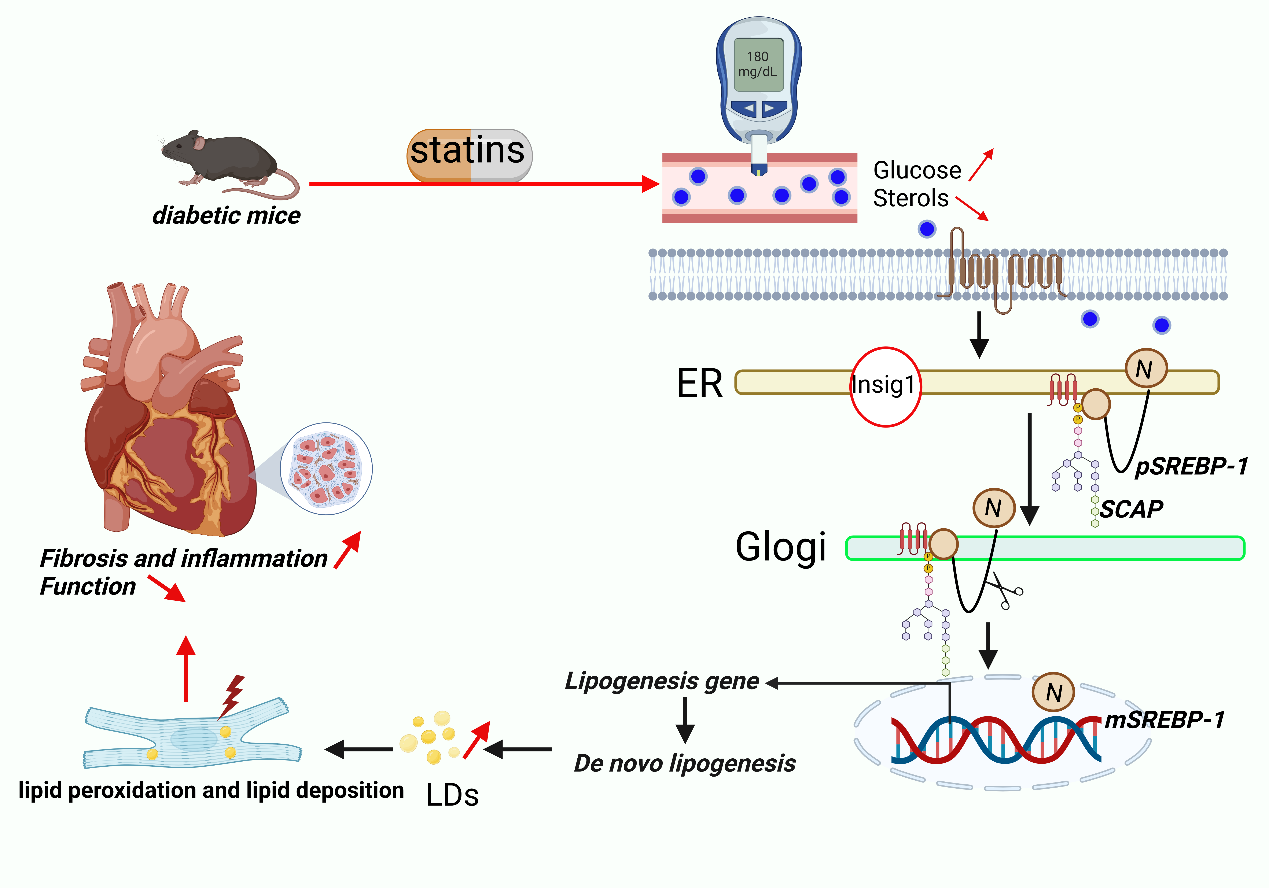


**Supplemental Figure 9.** **Schematic diagram illustrating SREBP1 induction mediates long-term statins therapy related myocardial lipid peroxidation and lipid deposition in TIIDM mice.**

Long-term statins administration elevated myocardial de novo lipogenesis via enhancing intracellular accumulation of glucose promotes SCAP N-glycosylation leading to SREBP-1 activation, which promoted myocardial lipid peroxidation and lipid deposition to myocardial dysfunction in TIIDM mice. Graphics were created with Biorender.com.
